# On-Skin Epidermal Electronics for Next-Generation Health Management

**DOI:** 10.1007/s40820-025-01871-5

**Published:** 2025-08-08

**Authors:** Jinbin Xu, Xiaoliang Chen, Sheng Li, Yizhuo Luo, Shizheng Deng, Bo Yang, Jian Lv, Hongmiao Tian, Xiangming Li, Jinyou Shao

**Affiliations:** 1 Frontier Institute of Science and Technology (FIST), Xi’an Jiaotong University, Xi’an, 710049 Shaanxi People’s Republic of China; 2 State Key Laboratory for Manufacturing Systems Engineering, Xi’an Jiaotong University, Xi’an, 710049 Shaanxi People’s Republic of China; 3 Interdisciplinary Research Center of Frontier Science and Technology, Xi’an Jiaotong University, Xi’an, 710049 Shaanxi People’s Republic of China

**Keywords:** On-skin epidermal electronics, Adhesiveness, Breathability, Mechanoelectrical stability, Long-term biosignal monitoring

## Abstract

Continuous monitoring of biosignals is essential for advancing early disease detection, personalized treatment, and health management. Flexible electronics, capable of accurately monitoring biosignals in daily life, have garnered considerable attention due to their softness, conformability, and biocompatibility. However, several challenges remain, including imperfect skin-device interfaces, limited breathability, and insufficient mechanoelectrical stability. On-skin epidermal electronics, distinguished by their excellent conformability, breathability, and mechanoelectrical robustness, offer a promising solution for high-fidelity, long-term health monitoring. These devices can seamlessly integrate with the human body, leading to transformative advancements in future personalized healthcare. This review provides a systematic examination of recent advancements in on-skin epidermal electronics, with particular emphasis on critical aspects including material science, structural design, desired properties, and practical applications. We explore various materials, considering their properties and the corresponding structural designs developed to construct high-performance epidermal electronics. We then discuss different approaches for achieving the desired device properties necessary for long-term health monitoring, including adhesiveness, breathability, and mechanoelectrical stability. Additionally, we summarize the diverse applications of these devices in monitoring biophysical and physiological signals. Finally, we address the challenges facing these devices and outline future prospects, offering insights into the ongoing development of on-skin epidermal electronics for long-term health monitoring.

**Graphical abstract:**

## Highlights

- This review comprehensively examines representative functional materials, analyzes their intrinsic properties, and illustrates how rational structural design and fabrication strategies can be employed to achieve high-performance epidermal electronics.
- Three essential performance requirements for long-term, continuous health monitoring—adhesiveness, breathability, and mechanoelectrical stability—are emphasized, alongside effective strategies for their realization.
- Current scientific challenges in this field are critically discussed, offering in-depth insights into the development of next-generation on-skin epidermal electronics aimed at transforming personalized healthcare.

## Graphical abstract

**Figure:**
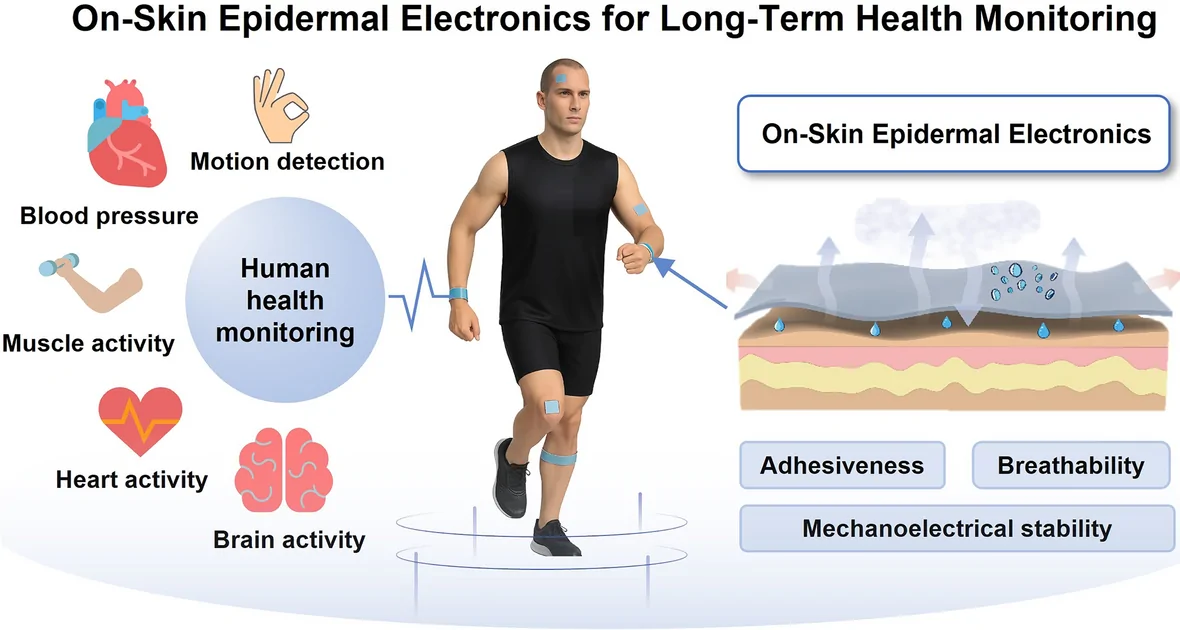

## Introduction

The continuous monitoring of physiological signals is of pivotal importance in the advancement of healthcare. By providing real-time data on key biological parameters such as heart rate, blood pressure, or electrophysiological signals, long-term health monitoring enables early detection of diseases, personalized treatment, and effective health management [1–3]. This approach is particularly critical as the global population ages and the prevalence of chronic diseases increases, necessitating innovative solutions for proactive healthcare. The skin, as the largest organ of the human body, provides essential physiological signals indicative of health status [4, 5]. Recent advancements in functional materials, micro-nano manufacturing, and microelectronics technologies have led to the development of numerous commercial wearable devices, including smartwatches, fitness bands, and bioelectrodes. These devices can monitor human physiological signals such as body movement, heart rate, blood pressure, and temperature in daily life. However, current commercial products, due to their rigid hardware, encounter significant challenges, including poor skin conformity and motion artifacts, arising from mechanical mismatches between rigid devices and the soft, deformable skin. Moreover, the bulkiness and lack of self-adhesive properties of these devices limit their applicability to specific body locations, significantly restricting comfort and hindering the ability to measure a wide range of physiological signals.

In recent years, flexible, wearable electronics fabricated on thin, flexible polymer substrates have emerged as promising alternatives [6–8]. Characterized by their softness and stretchability, these devices can conformally adhere to the skin, enabling comfortable and versatile monitoring of physiological signals [9, 10]. With excellent biocompatibility, robust mechanical properties, and high integration potential, flexible wearable electronics have become ideal platforms for health monitoring [11, 12], drug delivery [13], and other biomedical applications [14, 15], as well as emerging technologies such as human–machine interaction [16] and the Internet of Things [17]. Despite significant progress in wearable electronics, challenges persist in interfacing these devices with human skin. While current flexible wearable devices exhibit a certain degree of flexibility and can achieve macroscopic skin conformal contact, mechanical mismatches between the flexible substrates and skin still hinder perfect contact and identical deformation of the sensors [18]. At the microscopic scale, the irregular and hairy surface of the skin further results in insufficient interface contact between skin and wearable devices [19, 20]. Untight contact and imperfect interfaces can cause relative movement between the device and skin, or even detachment, which gives rise to persistent motion artifact issues and inaccurate signal detection [21]. Moreover, most current flexible wearable devices lack self-adhesiveness and require additional tapes or adhesives for attachment to the skin. Both dense film substrates and adhesive tapes limit the breathability and deformability of the skin and cause perspiration build-up, restricting their applications in long-term and comfortable physiological signal monitoring.

On-skin epidermal electronics represent a type of thin, soft, and lightweight wearable electronic device that can conform seamlessly to the skin surface without hindering skin deformation [22–24]. The seamless, comfortable, robust skin-device contact interface and long-term mechanoelectrical stability distinguish on-skin epidermal electronics from other forms of wearable electronics. Owing to the unique characters, the on-skin epidermal electronics become an ideal candidate for long-term and continuous health monitoring that can accurately capture critical bio-signals and reflect real-time human health information. They operate by transducing physiological signals such as pressure, strain, temperature, and electrophysiological potentials into measurable electrical outputs. For instance, electrophysiological sensors (e.g., electrocardiogram (ECG), electromyogram (EMG), and electroencephalogram (EEG)) detect biopotentials generated by ionic flows in tissues via skin-contact electrodes, converting them into electronic signals for interpretation [25]. Similarly, piezoresistive/thermoresistive, piezoelectric/thermoelectric, capacitive or triboelectric sensors respond to mechanical deformation, pressure or temperature fluctuations through changes in resistance, capacitance or electric outputs, enabling real-time monitoring of human motion [26]. These sensing mechanisms allow for high-resolution, continuous health data acquisition in a minimally invasive manner, thus serving as an effective bridge between human physiology and digital diagnostics. Numerous reviews on wearable flexible electronics have been conducted, with some focusing on the material selection [27] and structural design [28] of specific sensors, such as strain [29] and pressure sensors [30], while others concentrate on the use of specific manufacturing technologies [31] in wearable sensors or the monitoring of specific diseases [32]. However, there is a paucity of reviews addressing the construction of high-performance epidermal electronic components through rational selection of functional materials and structural design, especially focusing on the attributes required to achieve long-term and continuous high-fidelity health monitoring. Consequently, a comprehensive review focusing on achieving long-term, comfortable, and stable bio-signal monitoring with epidermal electronics is imperative to promote the advancement of wearable medical technology.

**Fig. 1:**
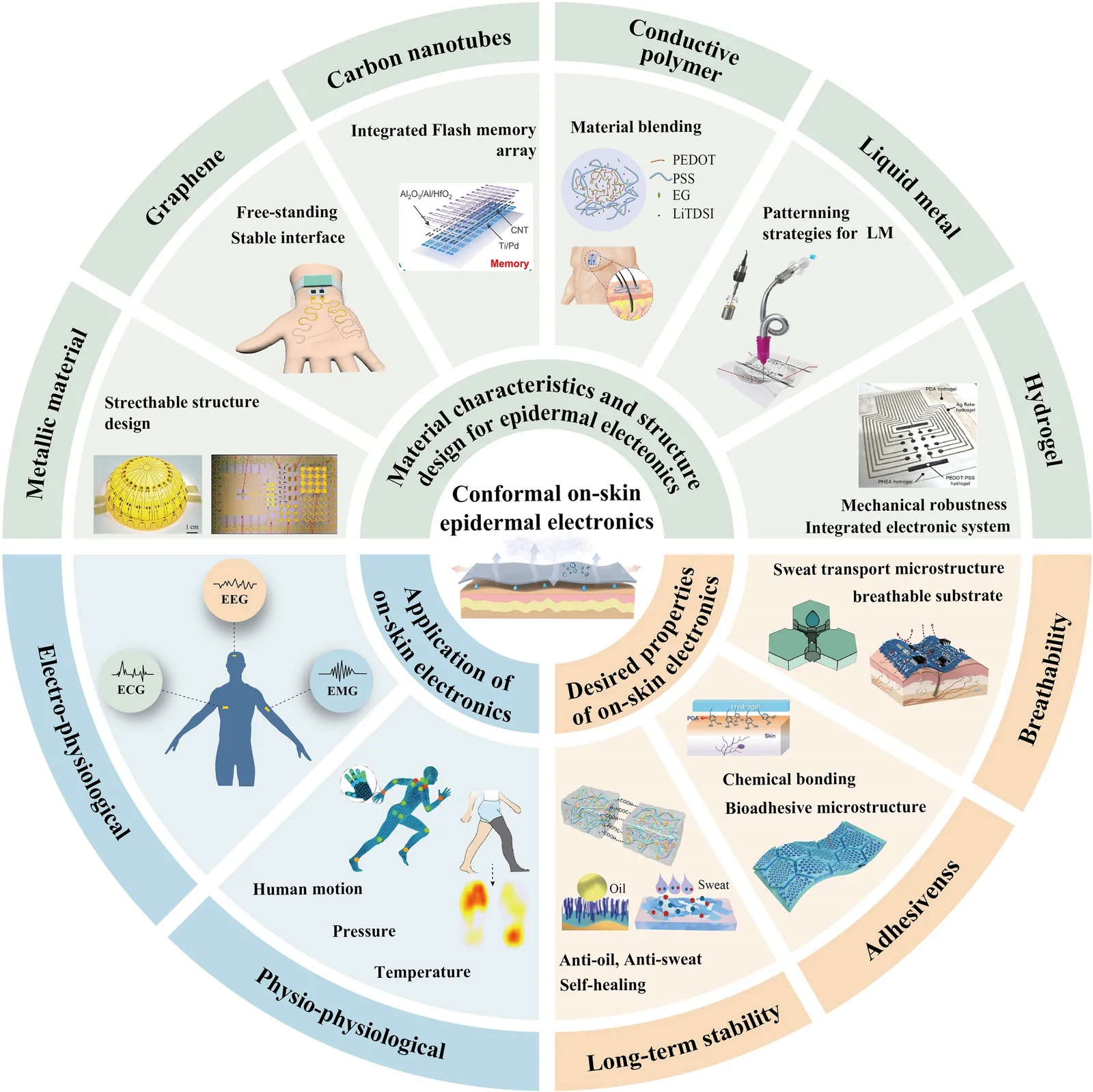
Overview of the on-skin epidermal electronics with desired properties based on different functional materials and structure design for physiological signals monitoring. Metallic material, Reproduced with permission [35]. Copyright 2013, Wiley–VCH. Reproduced with permission [39]. Copyright 2021, Wiley–VCH. Graphene, Reproduced under terms of the CC-BY license [59]. Copyright 2022, Springer Nature. Carbon nanotubes, Reproduced with permission [67]. Copyright 2022, American Association for the Advancement of Science. Conductive polymer, Reproduced with permission [79]. Copyright 2023, Elsevier. Liquid metal, Reproduced with permission [81]. Copyright 2024, American Association for the Advancement of Science. Hydrogel, Reproduced with permission [111]. Copyright 2024, Elsevier. Breathability, Reproduced with permission [136]. Copyright 2024, Springer Nature. Reproduced with permission [140]. Copyright 2024, Wiley–VCH. Adhesiveness, Reproduced with permission [117]. Copyright 2017, Wiley–VCH. Reproduced with permission [126]. Copyright 2019, Wiley–VCH. Long-term stability, Reproduced with permission [144]. Copyright 2021, American Association for the Advancement of Science. Reproduced with permission [145]. Copyright 2020, Wiley–VCH. Reproduced with permission [146]. Physio-physiological, Reproduced with permission [150]. Copyright 2023, Wiley–VCH. Reproduced with permission [153]. Copyright 2024, American Chemical Society

We begin by introducing the unique properties of diverse functional materials, followed by their structural engineering design and fabrication strategies for achieving high-performance epidermal electronics. Subsequently, we elaborate on the desired properties of on-skin epidermal electronics required for long-term and continuous health monitoring and the corresponding strategies for realizing them. Following this is the demonstration of prospective health monitoring applications for various on-skin epidermal electronics (Fig. 1). Finally, we discuss the scientific challenges of current electronics and provide perspectives for the development of on-skin epidermal electronics to revolutionize personalized healthcare technology. This review aims to promote the development of high-performance, lightweight, and breathable wearable epidermal electronics, attract researchers’ attention to long-term monitoring epidermal electronics, and accelerate the application of wearable on-skin epidermal electronics in daily healthcare fields.

## Material and Structure Design for On-Skin Epidermal Electronics

As previously mentioned, the complex microstructure of the skin presents significant challenges in achieving optimal contact with wearable devices and accurately acquiring physiological signals. Furthermore, to achieve comfortable and long-term health monitoring, on-skin electronics must be soft and breathable. To achieve these goals, selecting appropriate materials and implementing an optimal structural design are crucial, as these factors directly impact the sensing performance and wearing comfort of the device. This section provides an overview of various functional materials and corresponding structural designs that enable the long-term application of on-skin epidermal electronics.

### Metallic Materials

**Fig. 2:**
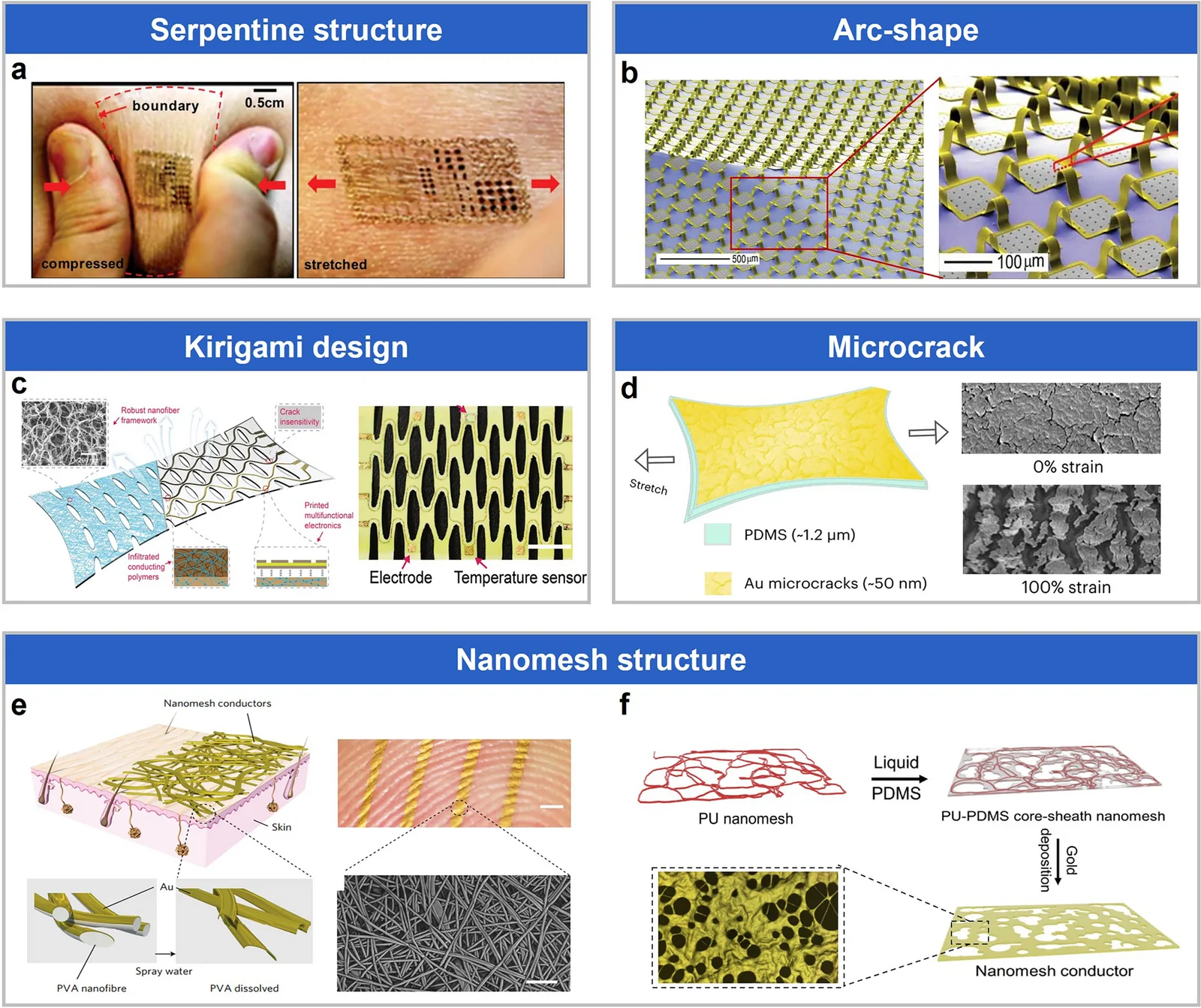
Metal-based on-skin electronics. **a** Injection-based epidermal electronic system with serpentine-structured, stretchable conductors. Reproduced with permission [35]. Copyright 2011, American Association for the Advancement of Science. **b** Photographs of arc-shaped metal interconnects circuit mesh. Reproduced with permission [37]. Copyright 2009, Wiley–VCH. **c** Composite nanofiber framework-based Kirigami membrane with integrated multifunctional electronics. Reproduced with permission [40]. Copyright 2022, Wiley–VCH. **d** A 1.3-µm-thick elastic conductor with a controlled morphology of microcracks in the gold film. Reproduced with permission [41]. Copyright 2022, Springer Nature. **e** Stretchable Au nanomesh epidermal electronics. Reproduced with permission [43]. Copyright 2017, Springer Nature. **f** PU nanofibers-PDMS core-sheath nanomesh conductors. Reproduced with permission [44]. Copyright 2020, American Association for the Advancement of Science

Metallic materials like gold and silver exhibit superior electrical properties (~ 10^7^ s m^−1^) and chemical stability, enabling efficient and reliable signal transmission with minimal noise over extended periods. Additionally, mature fabrication techniques, including vacuum deposition and photolithography allow precise patterning of metallic films for complex device architectures, making them frequently employed as functional materials for epidermal electronics [33]. However, metals are inherently hard and brittle, which limits their ability to withstand strains exceeding 5%, severely constraining their applicability in wearable electronics. To impart sufficient stretchability to metallic materials for application in wearable epidermal electronics, researchers have proposed specific structural designs [34]. In a pioneering study, Kim et al*.* reported a filamentary serpentine structure of stretchable metallic epidermal electronics with a stretchability of approximately 30%, which could achieve mechanical matching with the skin (Fig. 2a) [35]. Subsequently, researchers construct various mechanical models to predict the stretchability and effective module of serpentine structure to instruct the design of the geometric structure [36] Similar structures, such as the arc-shaped (Fig. 2b) [37], 3D helical [38], Kirigami (Fig. 2c) [39, 40], and microcrack structures (Fig. 2d) [41], have also demonstrated the capacity to augment the tensile attributes of metallic materials, rendering them suitable for epidermal electronics.

Additionally, nanomesh structures and metallic nanowires (AuNWs, with a sheet resistance of 40–60 Ω sq^−1^) [42] are frequently employed in constructing stretchable epidermal electronics, offering superior electrical conductivity and ductility. Figure 2e shows the fabrication of an Au nanomesh based on electrostatic spinning technology [43]. The deposition of Au films on polyvinyl alcohol (PVA) fibers, followed by the application of the resulting structure directly to human skin, yield a tightly adhered Au nanomesh structure with excellent stretchability up to 40%. This structure holds potential for comfortable and permeable monitoring of human signals. To enhance the sensor’s durability, Wang et al*.* developed a PU-PDMS-based metal nanomesh, which offered excellent sustainability and durability (60% strain for 5000 cycles). The thin and soft nanomesh is capable of monitoring human motion without interfering with the natural movement of the skin (Fig. 2f) [44]. In addition, Zhu et al*.* reported a highly soft and fully conformal-contact nanomesh epidermal electrode through simultaneous conduction of electrospinning of polyamide and electrospraying of silver nanowires (AgNWs, with a sheet resistance of 4.14 Ω sq^−1^) [45]. The epidermal electrode exhibits a thickness of 125 nm, approximately 50% stretchability and only 1.2% electric resistance variation even after 50,000 bending cycles. It can be seen that the combination of electrospinning technology and metallic materials presents a promising strategy for constructing high-performance epidermal electronics [46–48]. Given the maturity of industrial-scale electrospinning, particularly needleless systems capable of continuous nanofiber production, nanomesh structure are, in principle, compatible with scalable manufacturing. However, their broader commercialization remains constrained by the high cost of noble metals such as gold and the reliance on vacuum-based deposition techniques, which collectively hinder cost-effective large-area production.

### Carbon Materials

Carbon materials are recognized as highly promising candidates for epidermal electronics due to their exceptional electrical conductivity and mechanical strength. In particular, low-dimensional carbon materials, including carbon nanotubes and graphene, are noteworthy for their high electrical conductivity, excellent flexibility, and lightweight characteristics, and non-toxicity relative to noble metals, making them suitable for the fabrication of flexible devices.

#### Graphene-Based On-Skin Epidermal Electronics

**Fig. 3:**
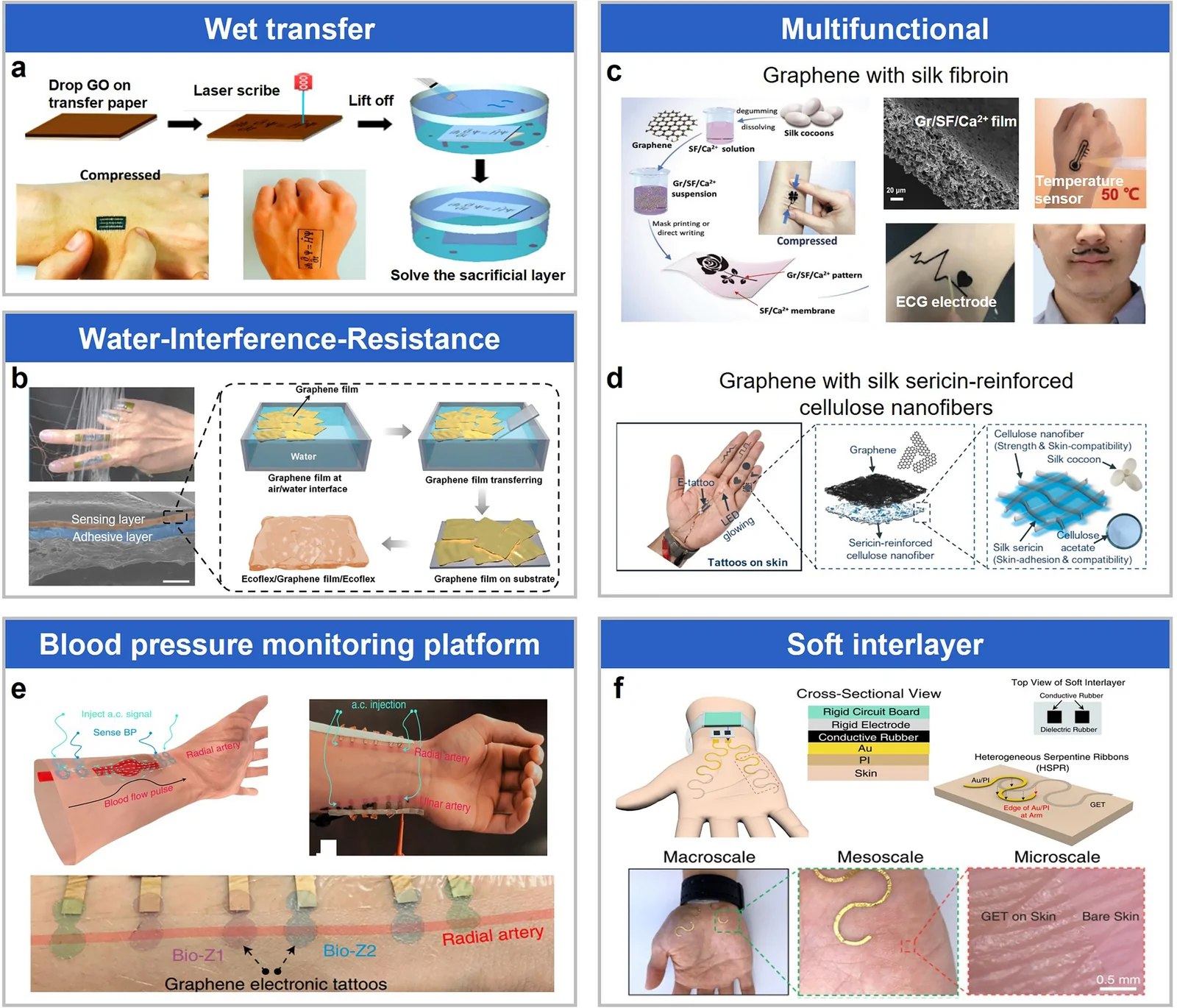
Graphene-based on-skin electronics. **a** Wet-transfer-graphene epidermal electronic patterned by laser reduction technology. Reproduced with permission [53]. Copyright 2018, American Chemical Society. **b** Highly adhesive graphene epidermal sensors with superior water-interference-resistance. Reproduced with permission [55]. Copyright 2023, Wiley–VCH. **c**, **d** Multifunctional epidermal electronics fabricated by hybrid material of graphene with different natural protein like silk fibroin (**c**) and silk sericin reinforced cellulose nanofibers (**d**). Reproduced with permission [56]. Copyright 2019, Wiley–VCH. Reproduced with permission [57]. Copyright 2024, Elsevier. **e** Graphene-based wearable blood pressure continuous monitoring platform. Reproduced with permission [58]. Copyright 2022, Springer Nature. **f** A wireless palm electrodermal activity (EDA) sensor based on GET connecting to a rigid EDA wristband through HSPR and a soft interlayer. Reproduced under terms of the CC-BY license [59]. Copyright 2022, Springer Nature

Graphene, with its two-dimensional honeycomb structure of carbon atoms, offers exceptional electron mobility (~ 10^8^ S m^−1^), high mechanical strength (tensile strength up to 130 GPa), and excellent flexibility, even at ultrathin thicknesses. These properties make graphene particularly suitable for transparent, lightweight epidermal electronics [49–51]. Despite its potential, graphene faces challenges in terms of large-scale production and patterning precision. To fabricate graphene electronic devices with desired pattern, good durability and compatibility with human skin, researchers proposed customizing the graphene pattern using dry patterning [52] or laser reduction technology (Fig. 3a) [53]. The patterned graphene epidermal electronics was then conformally applied to the human skin using wet transfer technology, enabling the measurement of physiological signals without interfacing with the skin’s natural deformation. However, graphene grown by chemical vapor deposition (CVD) are often costly and need complex transfer processes that may compromise its mechanical integrity and robustness [54]. To enhance the stability and resilience of graphene epidermal electronics in various complex scenarios (e.g., in aqueous scenarios), Wang et al*.* developed a self-adhesive epidermal sensor comprising an ultrathin Ecoflex-encapsulated interconnected graphene strain sensing layer and a semi-crosslinked poly(dimethylsiloxane) self-adhesive layer [55]. As shown in Fig. 3b, the graphene film is obtained at the air/water interface and subsequently transferred to an Ecoflex substrate, followed by encapsulated with another Ecoflex layer. This configuration enables the sensor to adhere securely to the skin (4.45 N m^−1^) even under extreme aquatic conditions (strong water impact up to 4 m s^−1^).

The combination of natural protein materials with graphene has the potential to facilitate the development of multifunctional, high-performance graphene-based epidermal electronics. For instance, Wang et al*.* mixed graphene with silk fibroin to formulate an ink to create patterned graphene epidermal electronics with self-healing properties by screen printing (Fig. 3c), enabling real-time monitoring of various signals, including strain, humidity, temperature, and ECG signals [56]. Not coincidentally, Joshi put forth self-powered epidermal electronics by incorporating graphene with silk sericin-reinforced cellulose nanofibers (Fig. 3d), which could be utilized in a multitude of scenarios, including motion detection, temperature sensing, and thermal therapy [57]. To enable long-term continuous signal monitoring, Akinwande et al*.* developed a graphene-based wearable blood pressure monitoring platform (Fig. 3e). This platform is characterized by its ultrathin, self-adhesive, and lightweight properties, allowing for the monitoring of arterial blood pressure for over 300 min [58]. In practical applications, establishing a reliable connection between ultrathin epidermal electronics and external rigid, thick circuit boards are a significant challenge. Figure 3f illustrates a heterogeneous serpentine ribbons (HSPR) design based on graphene e-tattoo (GET) and Au/PI serpentine ribbon lamination. The elegant structure can minimize the strain concentration at the interface between the GET and the circuit board, thereby ensuring a reliable electrical connection (high intrinsic adhesion energy of 7.687 J m^−2^ between graphene and Au). This approach offers an effective strategy for enhancing the signal fidelity of ultrathin epidermal electronics [59].

#### Carbon Nanotubes-Based On-Skin Epidermal Electronics

**Fig. 4:**
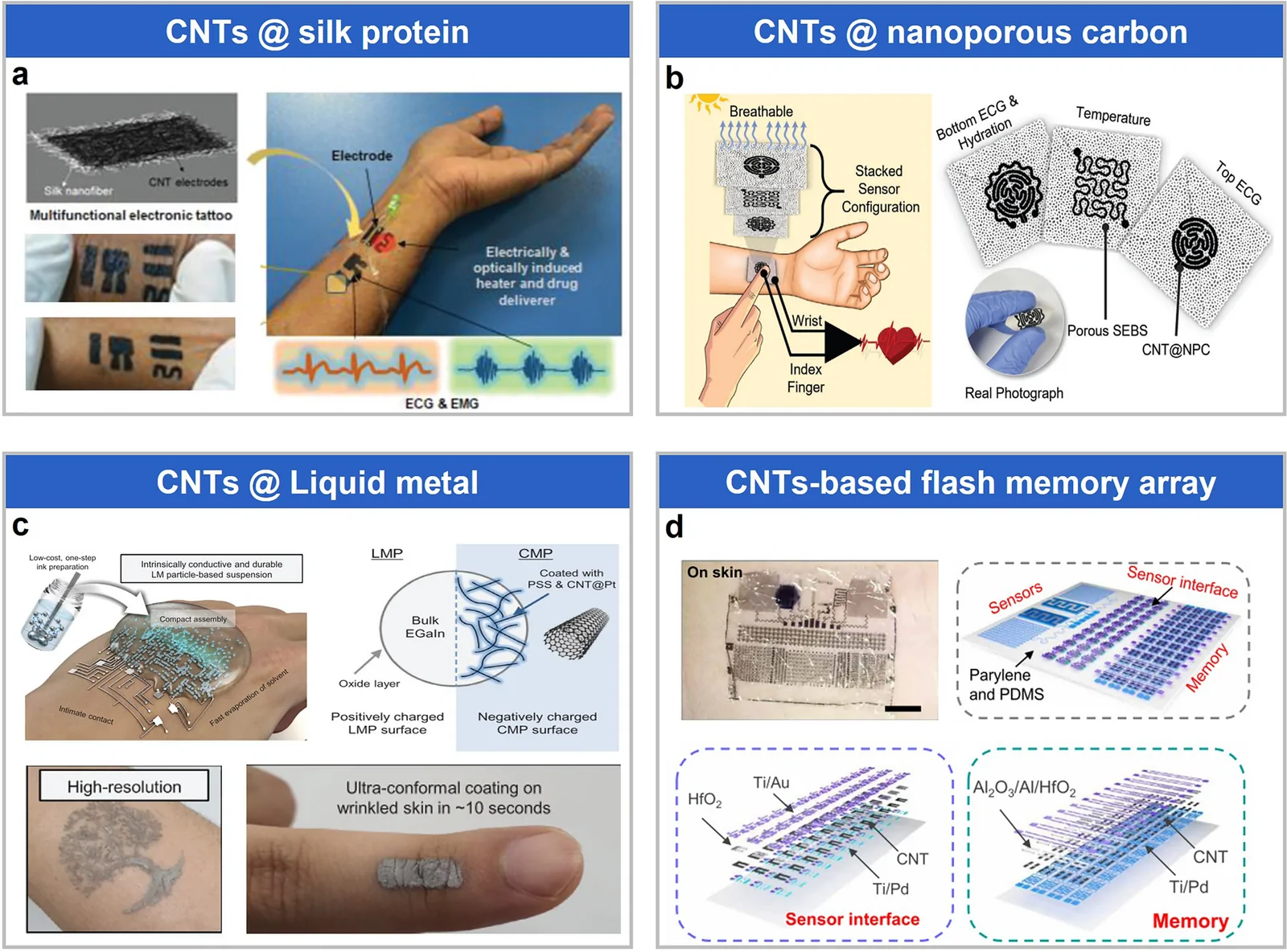
Carbon nanotubes-based on-skin electronics. **a** Multifunctional ultrathin CNT/SNF epidermal electronic systems. Reproduced with permission [63]. Copyright 2021, Wiley–VCH. **b** CNT@NPC ink-based strain-insensitive and breathable epidermal electronics. Reproduced with permission [64]. Copyright 2024, Wiley–VCH. **c** CNT-attached liquid metal particle-based conformal epidermal electronics. Reproduced with permission [66]. Copyright 2022, Wiley–VCH. **d** CNT-based differential amplifiers and flash memory array and integrated epidermal electronic system. Reproduced with permission [67]. Copyright 2022, American Association for the Advancement of Science

The structure of carbon nanotubes (CNTs) can be conceptualized as the arrangement of carbon atoms into a cylindrical lattice structure with single or multiple walls [60]. Carbon nanotubes have a very high aspect ratio (micrometer length and nanometer diameter), which provides an efficient pathway for the transfer of electrons and heat conduction. Consequently, carbon nanotubes exhibit electrical (~ 10^5^ s m^−1^) and thermal conductivity (6000 W m^−1^ K^−1^), making them suitable for diverse flexible electronics [61]. CNTs also exhibit good mechanical performance (tensile strength: 50–200 GPa, maximum bending strain: 18%), chemical stability and can be functionalized to improve their biocompatibility for skin applications. By combining highly conductive CNTs with porous silk protein nanofiber (SNF), Gogurla et al*.* proposed self-powered epidermal electronics that could be conformally adhered to human skin for the monitoring of human movement [62], and multifunctional ultrathin epidermal electronic systems that could be used for real-time monitoring of electrical signals, temperature sensing, and drug delivery (Fig. 4a) [63].

CNTs can be combined with other materials to create high-performance conductive inks. Figure 4b displays a combination of CNTs and nanoporous carbon (CNT@NPC) inks with molecular tuning capacity and superior conductivity (~ 1.5 × 10^–3^ Ω m), which can be applied to construct multifunctional, breathable, and strain-insensitive epidermal electronics through mask spraying technology. The CNT@NPC epidermal device is capable of acquiring real-time hydration, temperature, and physiological electrical signals from the human skin [64]. The combination of CNTs and liquid metal represents another potential avenue for achieving on-the-spot assemble and durable inks [65]. Figure 4c presents an electronic tattoo ink composed of liquid metal and CNTs, which exhibits excellent electrical conductivity (~ 1 kΩ sq^−1^) and mechanical stability. The ink enables swift and accurate pattern printing directly on the skin by a straightforward, single-step coating process, thereby facilitating personalized healthcare [66]. Furthermore, to construct the integrated epidermal electronic system through flexible devices, based on CNTs film, Xiang et al*.* developed an epidermal differential amplifier for weak signal processing and a flexible flash memory array for storing signals (Fig. 4d). The integration of these two components with physiological signal acquisition sensors enables the construction of an epidermal electronic system proficient in acquiring, processing, and storing biological signals. This research has further demonstrated the potential of epidermal electronic systems for the monitoring of physiological signals and the delivery of personalized medical diagnoses [67].

### Conductive Polymers

**Fig. 5:**
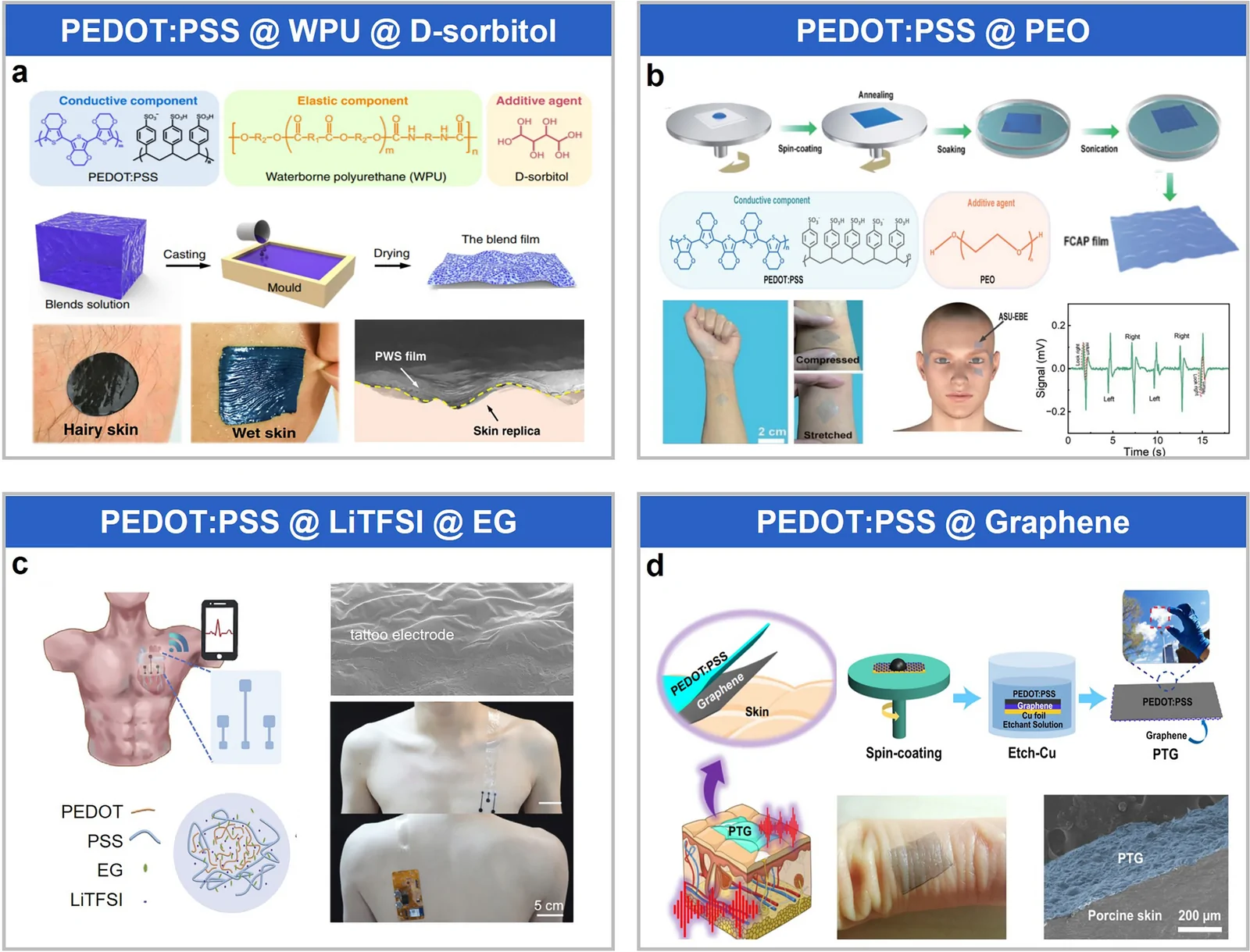
Conductive polymer-based on-skin electronics. **a** PEDOT:PSS/WPU/D-sorbitol self-adhesive dry electrode. Reproduced under terms of the CC-BY license [77]. Copyright 2020, Springer Nature. **b** Substrate-free breathable epidermal bioelectrodes for long-term physiological monitoring. Reproduced with permission [74]. Copyright 2024, Springer Nature. **c** Ultrahigh conductive PEDOT:PSS/ionic liquid epidermal tattoo. Reproduced with permission [79]. Copyright 2023, Elsevier. **d** Ultrathin (~ 1nm) and conformal skin electrodes with double-layer stacked structure of PEDOT:PSS/graphene. Reproduced under terms of the CC-BY license [80]. Copyright 2021, Springer Nature

Conductive polymers, including poly(3,4-ethylenedioxythio-phene):poly styrene sulfonate (PEDOT:PSS) [68], polyaniline (PANI) [69], polypyrrole (PPy) [70], and poly(3-hexylthiophene) (P3HT) [71], are also promising materials to construct on-skin epidermal electronics due to their mechanical flexibility, biocompatibility, and unique electronic-ionic conductivity. Among the existing conductive polymers, PEDOT:PSS is extensively utilized in flexible wearable electronic devices due to its adjustable electrical conductivity (< 0.0012 Ω cm) and biocompatibility [72]. Furthermore, its solution processability and molecular structure tunability allow researchers to fabricate epidermal electronics by various manufacturing processes, including screen printing [73], spin coating [74], 3D printing [75], and wet-spinning [76]. However, the poor stretchability and non-adhesive nature of pure PEDOT:PSS hinder their applications in high-performance epidermal electronics. Additionally, PEDOT:PSS has a lesser conductivity than metals or carbon materials, which restrict their use in situations where great signal fidelity is required. To overcome these challenges, Ou et al*.* developed a bio-dry electrode that is stretchable, self-adhesive, and highly conductive by combining PEDOT:PSS with waterborne polyurethane (WPU) and D-sorbitol (Fig. 5a). The blend film prepared by solution processing exhibits high electrical conductivity (> 390 S cm^−1^), excellent stretchability (elongation at break > 43%), remarkable self-adhesiveness and low skin–electrode impedance. The epidermal electronics can adhere firmly to dry or wet skin, even when subjected to stretching, deformation, vibration, and other forms of skin movements, making them suitable for acquiring high-quality epidermal biopotential signals under various skin conditions [77]. Similarly, Lai et al*.* proposed a substrate-free epidermal bioelectrode by incorporating water-soluble PEO into PEDOT:PSS, demonstrating excellent conductivity (475 S cm^−1^) and stretchability (~ 48%). The bioelectrode exhibits excellent breathability, with a rate sevenfold higher than that of typical percutaneous water loss (Fig. 5b) [74].

The incorporation of ionic liquids is an effective strategy to enhance the performance of the PEDOT:PSS-based electronics [78]. Xu et al*.* developed a conductive polymer system by incorporating ethylene glycol and bis(trifluoromethane) sulfonimide lithium salt (LiTFSI) into PEDOT:PSS (Fig. 5c). The fabricated epidermal electrode exhibits superior electrical conductivity (5165 S cm^−1^) and high tensile properties (elongation at break > 56.9%) and is capable of conformal attachment to human skin. The flexible epidermal electrode demonstrates minimal signal noise during static and dynamic monitoring and exhibits a lower skin impedance (10.6 kΩ cm^2^) at 100 Hz, which enables high-resolution real-time physiological signal monitoring [79]. Liu et al*.* designed a transparent, conductive, ultrathin dry electrode (100 nm) based on the synergistic interaction of CVD-grown large-area graphene films and PEDOT:PSS (Fig. 5d). The electrode exhibits extremely low surface resistance (24 Ω sq^−1^), high conductivity (4142 S cm^−1^), transparency, and electrical stability under tensile strain, which allows for a stable, continuous (12 h) and dynamic period of time to adhere to the skin in a conformal manner while maintaining a stable electrode–skin interface [80].

### Liquid Metal

**Fig. 6:**
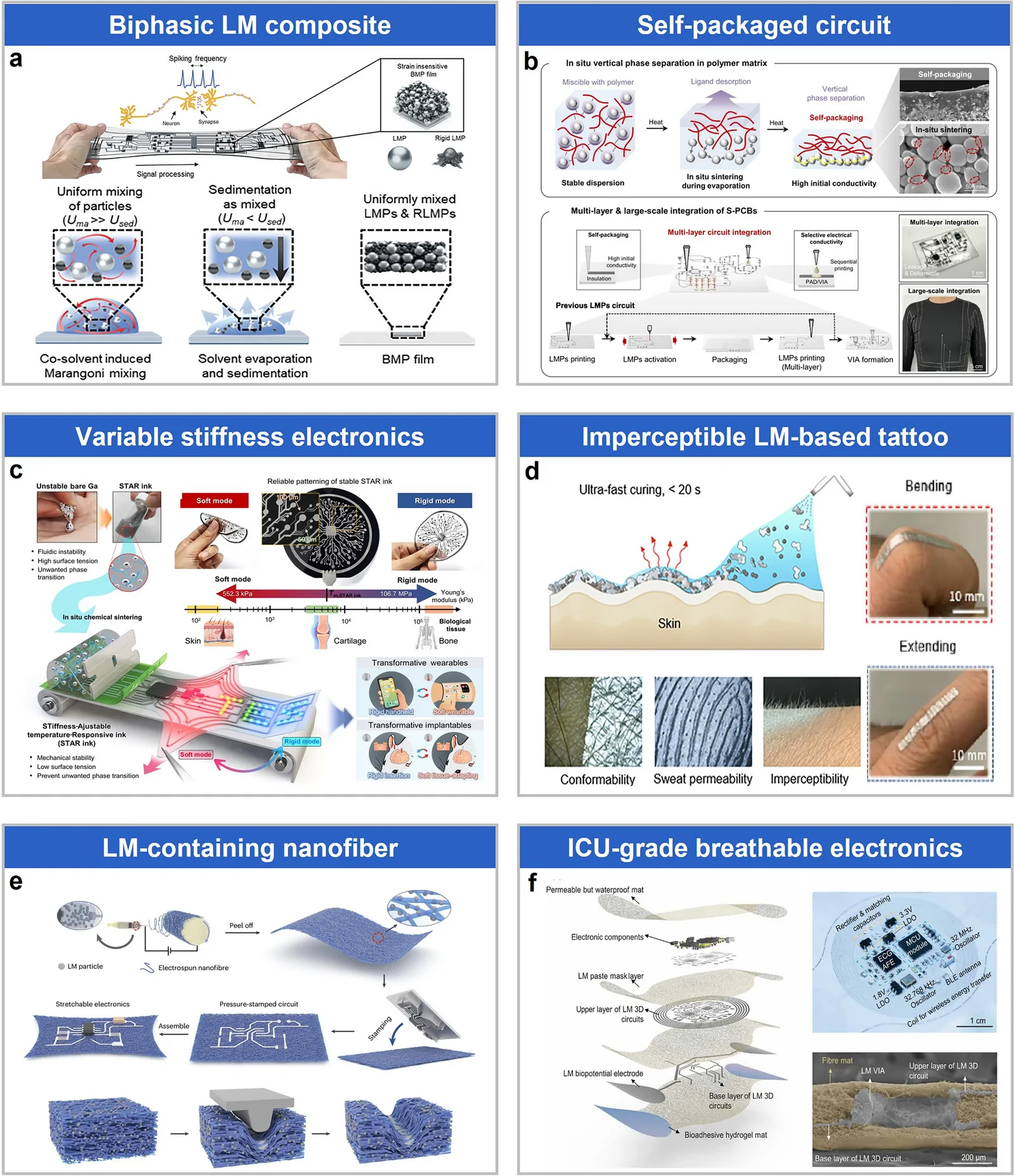
Liquid metal-based on-skin electronics. **a** Stretchable circuit with self-mixed biphasic liquid metal composite. Reproduced with permission [90]. Copyright 2024, Wiley–VCH. **b** Self-packaged stretchable printed circuits for multi-layer circuit and large-scale integration. Reproduced under terms of the CC-BY license [91]. Copyright 2025, Springer Nature. **c** Phase-change metal ink for fabrication of mechanically transformative electronics. Reproduced with permission [92]. Copyright 2025, American Association for the Advancement of Science. **d** Ultra-thin liquid metal tattoo on hairy skin. Reproduced with permission [93]. Copyright 2024, Elsevier. **e** Semi-embedded LM-particles nanofiber membrane for pressure-stamped stretchable electronics. Reproduced with permission [94]. Copyright 2024, Springer Nature. **f** LM-based ICU-grade breathable cardiac electronic skin. Reproduced with permission [95]. Copyright 2025, American Association for the Advancement of Science

Conventional metals and two-dimensional materials typically exhibit high Young’s modulus, limiting their applicability in highly stretchable electronic systems. In contrast, room-temperature gallium-based liquid metals (LMs) have recently attracted great interest due to their extraordinary attributes, including electrical conductivity (10^6^ s m^−1^), stretchability (500%), self-healing properties, and unique fluidity (the capacity to maintain electrical continuity under significant deformation). However, the inherently high surface tension of LMs presents a major challenge for their direct patterning in on-skin devices [81]. To address this, considerable research has focused on tuning the physicochemical properties of LMs, including rheological behavior [82], wettability [83] and interfacial adhesion [84] through strategies such as the application of mechanical forces [85], particle size reduction [86], compositional hybrid [87], or oxide surface modification [88]. One commonly adopted approach involves the formation of LM-based pastes by incorporating LM particles (LMPs) into polymeric matrices or blending them with conductive fillers. While this enhances processability, such composites often require external activation steps—such as laser ablation, mechanical rupture, or acoustic stimulation—to remove insulating barriers and restore conductivity [89]. These post-processing steps may introduce defects (e.g., open circuits), increasing manufacturing complexity and reducing reliability of large-area circuit. Moreover, many composites sacrifice electrical performance for better printability, which limits their use in high-performance interconnects. To overcome these challenges, Lee et al*.* developed a biphasic LM particle (BMP) composite by uniformly blending conventional LMPs with rigidified LMPs (RLMPs) (Fig. 6a). The resulting biphasic ink exploits spontaneous solutal-Marangoni mixing flows and subsequent gravitational settling processes to achieve high-resolution patterning without any post-treatment. It exhibits excellent stretchability and negligible resistance variation under strain (ΔR/R = 1.4 at 1200% strain), along with high conductivity (2.3 × 10^6^ S m⁻^1^), offering a robust solution for soft, deformable electronics [90].

Another challenge posed by the high fluidity of LMs is the risk of leakage, which necessitates reliable encapsulation. Traditional multilayer packaging methods, however, are often complex and incompatible with unconventional substrates such as textiles. Seo et al*.* proposed a novel concept of in situ sintering and self-packaging, whereby LMPs undergo phase separation within various polymer matrices during solution-based printing. This strategy allows for simultaneous pattern formation, high initial conductivity (8.75 × 10^6^ S m^−1^), and self-packaging within the polymer host, eliminating the need for external encapsulation (Fig. 6b). The resulting multilayer circuits show excellent mechanical durability (retaining conductivity after 10,000 stretching cycles at 100% strain), leak-proof performance, and potential for large-scale integration in bioelectronics [91]. Reconfigurable electronics with tunable stiffness are gaining interest for their ability to adapt to different mechanical demands during operation. Lee et al*.* developed a room-temperature processable gallium-based ink named as STAR (stiffness-adjustable temperature-responsive) ink by dispersing micro-sized gallium particles in a hydrophilic polyurethane (HPU) solution (Fig. 6c). STAR ink enables high-resolution patterning (~ 50 μm), 3D structural coating, and scalable manufacturing. Through pH-triggered chemical sintering, the resulting devices exhibit high conductivity (2.27 × 10^6^ S m⁻^1^), large stiffness tunability (up to ~ 1500 × in 600 μm-thick device), and rapid reversible transitions between soft and rigid states, paving the way for next-generation biomedical and soft robotic systems [92].

To translate LM conductors into wearable epidermal platforms, several patterning strategies have been explored. Yang et al*.* designed a conductive ink consisting of WPU, silver flakes, and liquid metal, which could be deposited directly to human skin onto form a patterned conductive pathway by mask spray coating (Fig. 6d). The prepared electronic tattoo can closely fit the skin and conform to the texture and microstructure of the skin while exhibiting excellent breathability and wearing comfort [93]. Additionally, LM integration into nanofibrous membranes has demonstrated promising gas permeability and mechanical resilience. Zheng et al. introduced a nanofiber-based LM composite membrane (LMNM), in which embedded LM particles rupture under pressure to form conductive networks within electrospun polymer fiber mats (Fig. 6e). Pressure-assisted circuit stamping enables fine feature resolution (~ 50 μm), excellent mechanical durability (> 30,000 cycles at 100% strain), high stretchability (up to 400%), and impressive water vapor transmission rates (WVTR) of 2941 g m^−2^ d^−1^, making it highly suitable for skin-interfaced electronics [94]. Further extending LM applications, Zhuang et al. developed a wireless, breathable, and fully integrated cardiac electronic skin system for continuous ICU-level cardiac monitoring (Fig. 6f). The device features multilayered, stretchable, and breathable LM microcircuits embedded within a soft matrix, enabling dense integration of electronic components with low interfacial impedance, soft adhesive interfaces, and biocompatible architecture. This system supports real-time wireless acquisition, analysis, and transmission of cardiac data, demonstrating potential for both clinical and daily health monitoring [95]. In summary, the unique combination of high conductivity, extreme deformability, and multifunctional integration offered by liquid metals establishes them as highly promising candidates for next-generation epidermal electronics, particularly in applications demanding seamless skin conformation, long-term stability, and complex mechanical adaptability.

### Hydrogel

**Fig. 7:**
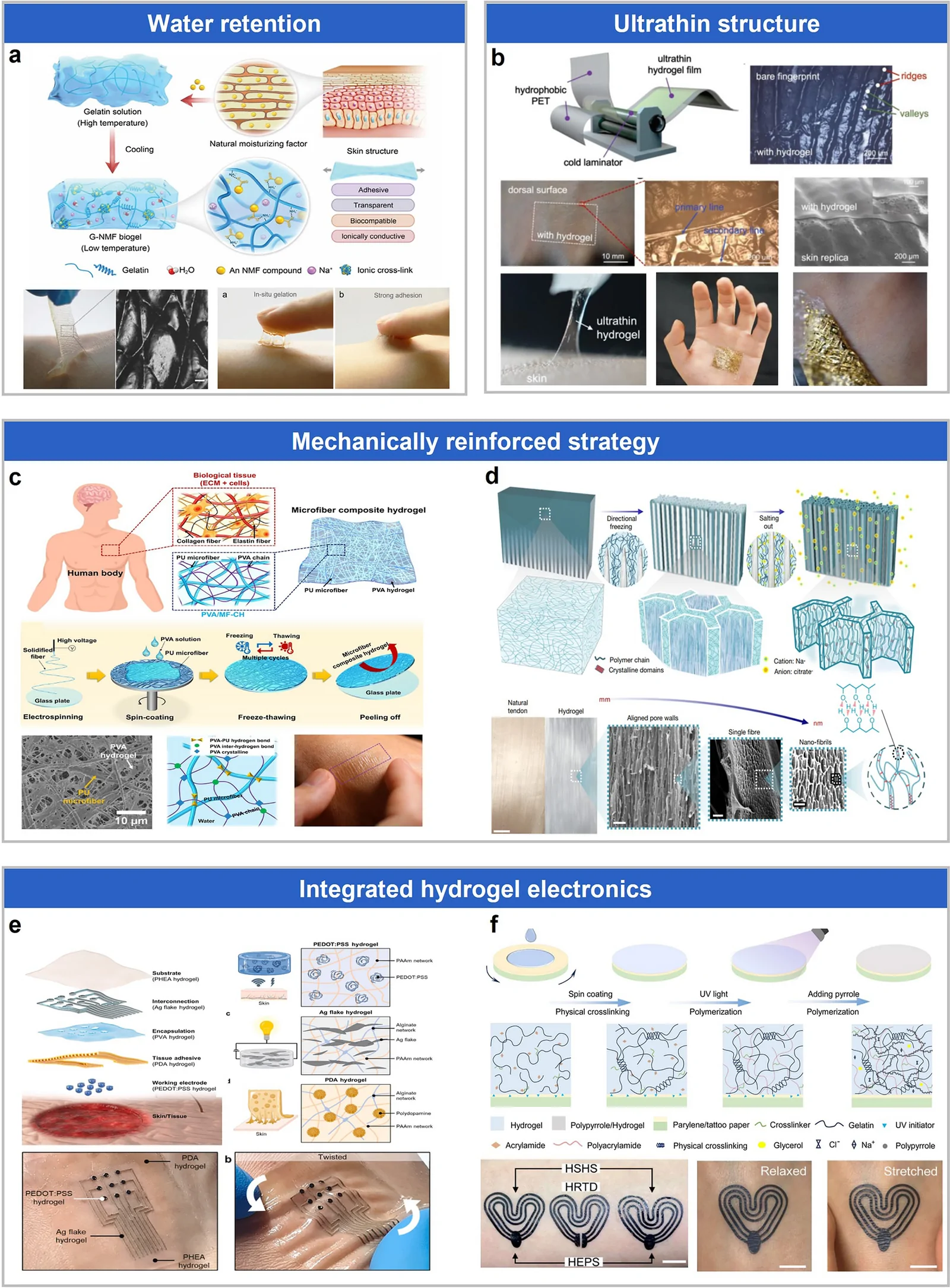
Hydrogels used for on-skin electronics. **a** In situ formed hydrogel-based epidermal electronics with high water retention. Reproduced with permission [103]. Copyright 2024, Wiley–VCH. **b** Illustration of ultrathin hydrogel films with high conformability and breathability. Reproduced with permission [104]. Copyright 2022, Wiley–VCH. **c** Ultrathin, breathable, and robust epidermal hydrogel electronics with nanofiber-reinforced structure. Reproduced under terms of the CC-BY license [105]. Copyright 2023, Springer Nature. **d** Freezing-assisted salting-out fabrication procedure and hierarchical structures for mechanically robust hydrogel fabrication. Reproduced with permission [109]. Copyright 2021, Springer Nature. **e** All-hydrogel-based multifunctional integrated electronics. Reproduced with permission [111]. Copyright 2024, Elsevier. **f** Multifunctional ultrathin hydrogel electronic tattoo for long-term signal monitoring. Reproduced with permission [112]. Copyright 2024, Springer Nature

Hydrogels are water-permeable, crosslinked polymer networks with tissue-like compliance (Young’s modulus in the range of 1–100 kPa), adjustable ionic conductivity, and excellent biocompatibility, and are extensively applied in the fields of tissue engineering [96, 97], biomedicine [98, 99], and flexible electronics [100]. The distinctive tissue-like mechanical properties of hydrogels facilitate the minimization of biomechanical mismatch and achieve stable adhesiveness at the skin–electrode interface [101]. Compared to dry electrode materials, the high-water content of hydrogels provides a wet and ion-rich physiological environment, enabling hydrogel electrodes to monitor physiological signals through the integration of electronic and ionic activity [25]. Furthermore, the exceptional versatility in the design of electrical, mechanical, and biological properties of hydrogels renders them a unique material for biological application. Nevertheless, a significant obstacle to the long-term utilization of hydrogels is their proclivity to dehydrate, resulting in the deterioration of their properties over time. To address this issue, Wang *et.al* proposed a non-drying zwitterionic skin by combining polyacrylic acid with glycerylphosphorylcholine (GPC), which can steadily uptakes 27.6 wt% water at RH 60% and remains stable for more than one month [102]. Additionally, the incorporation of glycerol or moisturizing factors into the into the hydrogel matrix can form more stable hydrogen bonds with water to lock in moisture and prevent dehydration, effectively enhancing the stability of the biogel. For instance, glycerol-modified gelatin hydrogels have been reported to retain approximately 70% of their initial water content after 100 h under ambient conditions [20]. Another study introduced the natural moisturizing factor named sodium pyrrolidone carboxylic acid (PCA-Na) into gelatin to confer it with anti-dehydration properties through the formation of hydrogen between PCA-Na and water (Fig. 7a). The biogel containing 40 wt% PCA-Na shows a dehydration-related weight reduction from 80% (in pure gelatin hydrogel) to less than 30%, attributed to both stable hydrogen bonding formation and lower vapor pressure of PCA-Na. Furthermore, the ionic cross-linking between the amino groups of gelatin chains and the carboxyl groups of PCA-Na significantly enhance the mechanical strength of the biogel [103]. In addition, the reversible fluid-gel transition property of gelatin enables the biogel to be in situ gelatinized on human skin with seamless and stable conformal contacts, which can be applied as a high-quality and long-term interface for recording physiological electrical signals [19].

While the in situ polymerized gels described above can establish stable interfaces, their thickness and uniformity are difficult to control due to the direct coating method, which affects the deformation and free breathing of the skin to a certain extent. Figure 7b displays a 10μm-thick, peelable ultrathin hydrogel fabricated by the cold lamination technique, which exhibits a Young’s modulus akin to that of human skin. The ultrathin structure allows the hydrogel to have excellent breathability (1890.0 ± 134.4 g m^−2^ d^−1^) and a seamless contact to the skin, presenting a simple solution for the integration of epidermal electronics [104]. Despite their excellent properties, micrometer-thick hydrogels are deficient in mechanical robustness and are susceptible to mechanical damage. To enhance the mechanical characteristics of ultrathin hydrogels while preserving their ultrathin attributes and breathability, Zhang et al*.* developed an ultrathin tissue-inspired microfiber composite hydrogel by embedding a PVA fiber network into the hydrogel (Fig. 7c). Despite its diminutive dimensions of 5 μm, the gel device demonstrates remarkable mechanical robustness, attributable to the reinforcing effect of the fiber network. Moreover, the modulus of the hydrogel can be adjusted by controlling the parameter of the fiber network, additives and thickness in a broad range to match the applications [105]. Although the high-water content in hydrogels makes them highly similar to extracellular matrices and ideal for tissue engineering, it also results in weak mechanical properties. A significant amount of research has been conducted with the objective of improving the mechanical strength of hydrogels [106–108]. For instance, hydrogels with an ultra-low solid content and good mechanical properties have been successfully synthesized by combining chain entanglement and peptide reinforcement methods. Peptide cross-linkers are employed in lieu of conventional cross-linkers to create a distinctive network of highly entangled hydrogels with exceptional mechanical properties [109].

Inspired by the structure of tendons, He et al*.* proposed a freeze-assisted salting-out strategy to improve the mechanical properties of hydrogels (Fig. 7d). This method modulates the hydrogel from both a molecular and structural engineering perspective, resulting in a multi-layered and anisotropic structure at multiple length scales, from the millimeter to the molecular level. This improves the complexity of the internal network, crystallinity, and density of the hydrogel, which greatly improves the toughness, strength, and fatigue resistance of the hydrogel [110]. Despite considerable advances having been made in the composition and structural design of hydrogels, the realization of hydrogel-based integrated bioelectronics remains a significant challenge. Shin et al*.* have achieved the development of all-hydrogel bioelectronics through the utilization of a variety of material designs of functionalized hydrogels and a stencil printing process, showing potential applications in the fields of bioimpedance monitoring, electric field stimulation, and drug delivery (Fig. 7e) [111]. Figure 7f shows a reusable 20 µm thick electronic tattoo based on a hydrogel with adjustable adhesion force, which is capable of achieving conformal contact with the skin. Furthermore, the sensor exhibits high water retention, enabling long-term and multimodal signal monitoring, including electro-physiological signals, skin hydration, and temperature, for up to six months [112].

In summary, the rational design of on-skin epidermal electronics requires a finely tuned balance between electrical conductivity**,** mechanical stretchability**,** and biocompatibility**.** These properties are inherently interdependent and often mutually constrained. For instance, metallic materials such as Au and Ag nanowires exhibit exceptional conductivity, yet their limited stretchability and potential cytotoxicity (for aquatic environments) restrict direct application on deformable skin surfaces. Carbon nanomaterials, including CNTs and graphene, offer enhanced flexibility and moderate conductivity, but face issues related to dispersion stability, aggregation, and long-term interface reliability. PEDOT:PSS represents a widely adopted conductive polymer with intrinsic biocompatibility; however, it exhibits humidity sensitivity and moderate conductivity unless doped or hybridized. Liquid metals provide an ideal combination of high conductivity and fluidic deformability, but their integration is challenged by high surface tension, leakage risks, and limited patterning precision. Hydrogels, owing to their intrinsic softness and biocompatibility, are ideal candidates for skin-interfacing electrodes, though they are limited by low electronic conductivity and susceptibility to dehydration. To overcome these material-level limitations, structural design strategies have emerged as a powerful complement to materials engineering. Geometrically engineered layouts, such as serpentine, mesh, or fractal architectures, endow intrinsically rigid conductors with mechanical compliance under strain. Composite approaches, where conductive fillers are embedded within stretchable elastomer matrices, enable stable percolation networks under dynamic deformation. Porous architectures, bilayer hybrids or nanofiber-reinforced hydrogels have also proven effective in enhancing toughness and mechanical integrity while preserving their bio-interface advantages.

Given these trade-offs, selecting an optimal material–structure combination requires quantitative comparison across key performance parameters. Table 1 provides a summary of representative material classes commonly used in epidermal electronics, highlighting their typical conductivity, stretchability, and biocompatibility levels, along with their practical applications.

**Table 1:** Summary of representative material and structure strategies for epidermal electronics

Materials	Structure/Fabrication strategies	Conductivity/resistance	Stretchability	Biocompability	Application	References
Au	Serpentine Kirigami	4.11 × 10^7^ S m^−1^	~ 30% ~ 60%	Good	Interconnecting electrodes,	[35] [39]
Au/PU-PDMS	Nanomesh	1.2 ± 0.36 Ω sq^−1^	~ 130%	Good	Strain mapping	[44]
Au/PDMS	Microcrack	6.33 × 10^−7^ Ω m	~ 300%	Good	Implantable neural interfaces	[41]
AgNWs	Electrospraying	4.14 Ω sq^−1^	~ 50%	Moderate (Ag⁺ toxicity)	ECG	[45]
Graphene	Laser scribing	1 kΩ sq^−1^	~ 10%	N/A	Strain sensor	[53]
Graphene/silk fibroin/Ca^2+^	Mask printing	N/A	~ 90%	N/A	Strain, humidity, temperature sensor	[56]
CNTs/silk nanofiber	Electrospinning	1.5 × 10^–3^ Ω m	N/A	12 h skin biocompatibility	Drug delivery, heater, ECG	[63]
CNTs/liquid metal	Mask printing	1 kΩ sq^−1^	N/A	5 days skin biocompatibility	Electrochemical Biosensors, EMG	[66]
PEDOT:PSS/WPU/D-sorbitol	Drop casting	> 390 S cm^−1^	~ 43%	Good	ECG, EMG, EEG	[77]
PEDOT:PSS/EG/ LiTFSI	3D printing	5165 S cm^−1^	~ 56.9%	Low risk of cytotoxicity	ECG, EMG, EEG	[79]
PEDOT:PSS/Graphene	Spin coating	4142 S cm^−1^	~ 40%	N/A	ECG, EMG, EEG	[80]
Biphasic liquid metal composite	Mask printing	2.3 × 10^6^ S m^−1^	~ 1200%	N/A	Stretchable interconnects	[90]
Liquid metal/ NMP/TPU	Screen printing	8.76 × 10^6^ S m^−1^	~ 215%	2 weeks in vivo biocompatibility	Multi-layered circuit, implantable devices	[91]
Ga particles/HPU/DMSO	Screen printing /dip coating	2.27 × 10^6^ S m^−1^	~ 1000%	Good biomechanical compatibility	Chronic neural interfacing	[92]
Gelatin/PCA-Na	Mold casting	25 mS cm^−1^	~ 256%	Good cytocompatibility	ECG, EMG, EEG	[103]
PAAm/alginate/Ag flake	Photopatterning	571.43 S cm^−1^	~ 270%	Good biocompatibility	EF stimulation, drug delivery	[111]

## Device Properties of On-Skin Electronics for Long-Term Health Monitoring

In addition to the rational selection of materials and structure design, it is essential to consider the mechanical compatibility and biocompatibility aspects between the epidermal electronics and the skin to ensure long-term and comfortable health monitoring. Specifically, a conformal and highly adhesive stable interface between the epidermal electronics and the skin is required. Furthermore, for long-term monitoring, the epidermal electronics must exhibit superior breathability and sustain stable and consistent sensing performance. In terms of these, this section discusses different design strategies that confer epidermal electronics with adhesiveness, breathability, and long-term mechanoelectrical stability.

### Adhesiveness

**Fig. 8:**
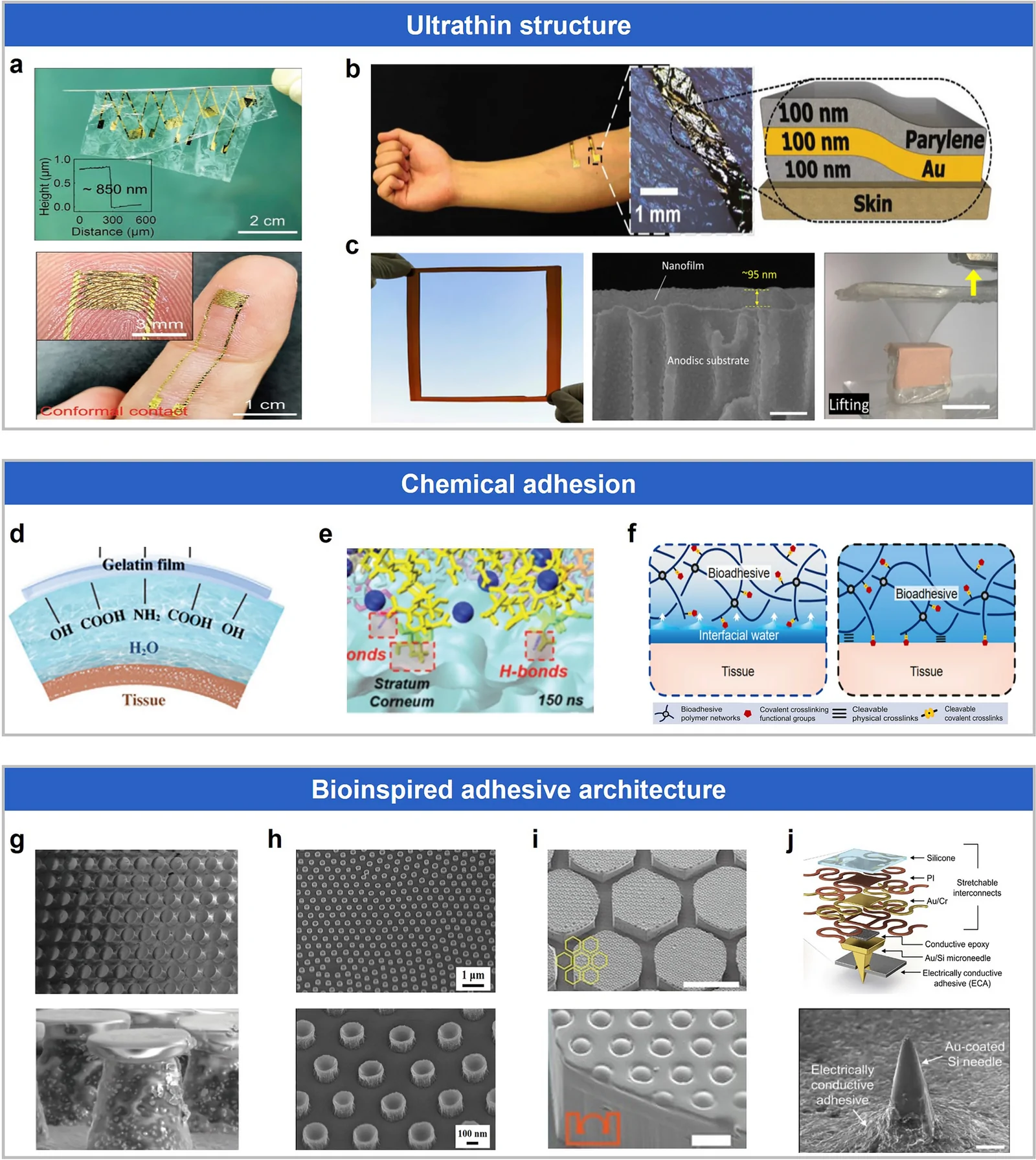
Strategies for realizing adhesiveness of on-skin electronics. **a** Ultrathin nano-engineered device with a thickness of 850 nm and its conformal attachment on a human’s fingertip. Reproduced with permission [114]. Copyright 2023, Wiley–VCH. **b** Skin-laminated sub-300 nm biopotential electrodes. Reproduced with permission [115]. Copyright 2018, Wiley–VCH. **c** Robust and highly adhesive 95 nm polyurethane–PDMS nanofilm. Reproduced under terms of the CC-BY license [116]. Copyright 2021, National Academy of Sciences. **d** Schematic of the hydrogen bonding between gelatin and stratum corneum (SC). Reproduced with permission [120]. Copyright 2024, Wiley–VCH. **e** Interaction between Alg-PAAm gel and SC. Reproduced with permission [121]. Copyright 2020, Wiley–VCH. **f** Dry cross-linking mechanisms of the bioadhesive for achieving instant tough adhesion. Reproduced under terms of the CC-BY license [122]. Copyright 2020, National Academy of Sciences. **g** Scanning electron micrography (SEM) image of micropillars structure electrode. Reproduced with permission [124]. Copyright 2018, Wiley–VCH. **h** Side-view SEM image of the octopus-inspired nanosucker array. Reproduced with permission [125]. Copyright 2017, American Chemical Society. **i** SEM images of the hierarchical structures of frog-inspired hexagonal microchannels and octopus-like convex structures for enhanced adhesivity. Reproduced with permission [126]. Copyright 2019, Wiley–VCH. **j** Structure design and micromorphology of stretchable microneedle adhesive patches. Reproduced with permission [128]. Copyright 2024, American Association for the Advancement of Science

A range of soft and stretchable on-skin epidermal electronics have been demonstrated to exhibit excellent skin compliance and sensing performance. However, these devices exhibit poor durability when attached to human skin for health monitoring due to the presence of hair on the surface of the human skin, which makes perfectly conformal attachment a significant challenge. The interface between the less adaptable on-skin epidermal electronics and the human epidermis is prone to gaps, resulting in an insufficiently robust interface. This makes the on-skin epidermal electronics incapable of withstanding the frequent, complex, and variable deformations of the skin. Moreover, the weak interface will result in a relative displacement between the epidermal electronics and the skin during movement, potentially leading to device detachment and a reduction in the fidelity of biosignal recording, which hinders the further application of epidermal electronics in long-term health monitoring. Consequently, researchers have endeavored to develop epidermal electronics with robust adhesion that can form a conformal contact with human skin. Ultrathin epidermal electronics can achieve full adhesion to the skin through Van der Waals forces, obviating the need for additional adhesives or tapes [21, 104]. Simultaneously, the ultrathin property can ensure the seamless contact between the epidermal electronics and wrinkled skin. Studies have indicated that devices with a thickness beyond 1.2 µm can cause discomfort and result in loss of conformal contact with microstructures possessed by human skin with a roughness of 30 µm [113]. In order to achieve ultrathin device fabrication, Liu et al*.* reported a nano-engineered ultrathin device using a dual sacrificial layer approach with a thickness of only 850 nm, which enabled perfect contact with human skin without requiring external adhesives (Fig. 8a) [114]. Figure 8b demonstrates a dry, thin-film electronic device with a thickness of under 300 nm (the thickness from 3.0 µm to 300 nm results in a dramatic increase of the average peel strength from ≈10.25 to 135.09 mN cm^−1^), which can be self-adhesively adhered to human skin to monitor physiological signals for up to 10 h [115]. Conformal and intimate contact with the skin requires that soft electronic devices endure significant deformations analogous to those of the skin. However, ultrathin thickness often compromises the mechanical properties of the device, thereby limiting the applicability of ultrathin electronics in long-term health monitoring. Consequently, it is important to develop new strategies to achieve a balance between mechanical performance and ultrathin properties. Figure 8c presents an ultrathin epidermal electronic device enhanced by nanofibers with a thickness of only 165 nm. The device exhibits exceptionally mechanical durability (tensile stress up to 7.82 MPa) and is capable of adhering to the skin with high skin adhesion energy (159 μJ cm^−2^) for up to one week of ECG monitoring [116].

In addition to ultrathin design, epidermal electronics can form stable interface with human skin through chemical bonds, including hydrogen bonds, electrostatic interactions, or covalent bonds. By interacting with the abundant chemical groups contained in the epidermis (like hydroxyl (–OH), carboxyl (–COOH), amine (–NH_2_), and amide (–CO–NH)), the resulting chemical skin-device interface exhibits stronger adhesion than that of van der Waals force interface [117–119]. As Fig. 8d shows, gelatin films are capable of forming hydrogen bonds through -OH, -COOH, and -NH_2_ interactions, thereby achieving adhesion to skin without affecting the morphology and function of the tissue [120]. Pan et al. reported a flexible self-adhesive electrode based on alginate-polyacrylamide (Alg-PAAm). The electrode can form a seamless and tight adhesion (90 N m^−1^) through strong electrostatic interactions and abundant hydrogen bonding with the skin, enabling the recording of weak surface EMG signals (Fig. 8e) [121]. Figure 8f displays a bioadhesive capable of forming fast and firm adhesion (5 s) on various wet tissues. The formation of physical and covalent cross-links with the wet tissue surface enables the creation of a long-term stable and high-strength adhesion (400 J m^−2^). This bioadhesive-electrode interface has the capacity to acquire ECG signals for up to 14 days. Therefore, the electrode is applicable to a wide range of materials and surface topologies, which has the potential to inform the future development of bio-integrated electronics for long-term monitoring [122].

Stable adhesion between epidermal electrons and the skin can also be achieved through the design of microadhesion structures [123]. For instance, Stauffer et al*.* put forth the concept of grasshopper feet-inspired micropillar electrodes (Fig. 8g), which were capable of attaining stable and precise conformal attachment to the surface of human skin (with a force up to 0.1 N cm^−2^) through augmented Van der Waals interactions [124]. However, this structure is unable to maintain sufficient adhesion in wet environments. Chen et al*.* proposed an octopus-inspired nanosuckers array, which was able to achieve multiple adhesion on both dry (3.0 N cm^−2^) and wet surfaces (2.8 N cm^−2^) through the adhesion generated by Van der Waals forces and the negative pressure effect, thereby extending the application scenarios of micro structured electrodes (Fig. 8h) [125]. Furthermore, by integrating the microchannel network observed in tree frog toe pads with the convex cup structure seen in octopus’ suckers, Kim et al*.* developed a hexagonal micropatterned hierarchical architecture electrode with enhanced pull adhesion and all-round peel resistance, thereby achieving high-strength pulling (max. 6.6 N cm^−2^ in dry conditions, 5.3 N cm^−2^ in moist conditions, and 4.5 N cm^−2^ in underwater conditions) and peeling direction (max. 26.8 J m^−2^ in dry conditions, 23.9 J m^−2^ in moist conditions, and 23.9 J m^−2^ in underwater conditions) (Fig. 8i) [126]. It is demonstrated that the exceptional adhesive properties could be attributed to the suction effect produced by the effective management of water residues and the enhanced crack inhibition. Microneedle patches have been demonstrated to exhibit reliable adhesive properties through mechanical interlocking with human skin [127]. Nevertheless, it has been demonstrated that the existing microneedle electrodes lack the requisite responsive elasticity. In light of the consideration, Kin et al*.* proposed a reliable microneedle adhesive patch with excellent stretchability. As illustrated in Fig. 8j, the soft and stretchable serpentine interconnect structure is designed to provide sufficient tissue compliance. The Au-coated silicon microneedle array can be inserted into the skin to achieve mechanical interlocking, and the conductive binder consisting of Ag flakes and high-tack silicone ensured firm adhesion between the device and the skin. This configuration is demonstrated to be capable of achieving reliable and long-term monitoring of electrophysiological signals [128].

### Breathability

**Fig. 9:**
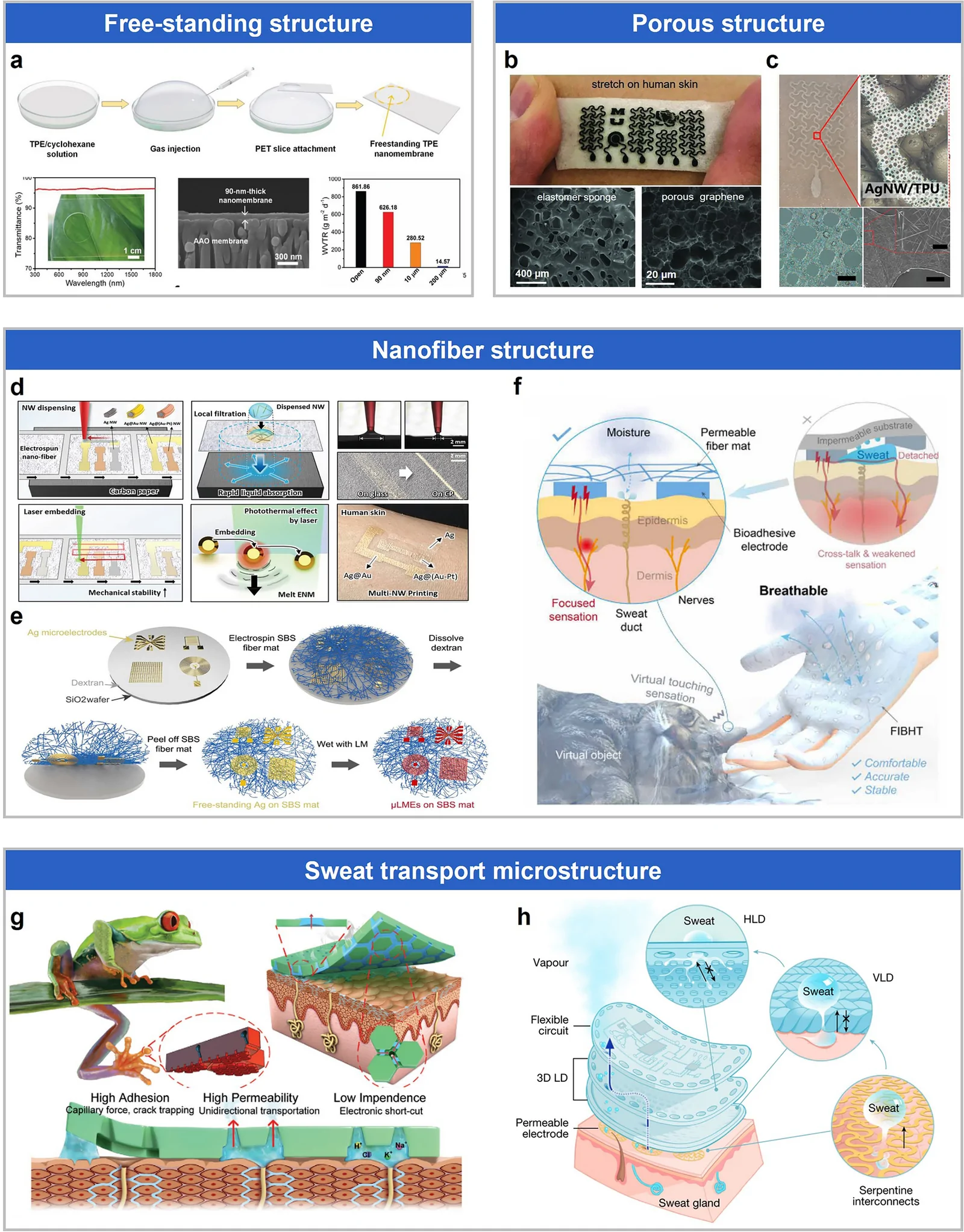
Strategies for realizing breathability of on-skin electronics. **a** Ultrathin epidermal electronics based on a facile bubble blowing method with excellent breathability. Reproduced with permission [131]. Copyright 2020, Wiley–VCH. **b** Breathable on-skin bioelectronic sensing systems using porous graphene and porous elastomeric sponge substrate. Reproduced with permission [134]. Copyright 2018, Wiley–VCH. **c** Porous TPU/AgNWs conductive film with high gas-permeability fabricated by the breath Figure method. Reproduced with permission [135]. Copyright 2020, American Chemical Society. **d** Local filtration-based nanowire printing process on electrospun nanofiber membrane. Reproduced with permission [137]. Copyright 2024, Wiley–VCH. **e** Fabrication process of permeable and high-resolution liquid metal microelectrode. Reproduced with permission [138]. Copyright 2023, American Association for the Advancement of Science. **f** Schematic of a fully integrated breathable haptic textile system. Reproduced with permission [139]. Copyright 2024, American Association for the Advancement of Science. **g** Treefrog-inspired wet-like electrode with beak-like asymmetric conical holes for enhanced permeability. Reproduced with permission [140]. Copyright 2024, Wiley–VCH. **h** Schematic of the integrated system-level sweat-permeable electronics, consisting of permeable electrodes, 3D LD (three-dimensional liquid diode with spatially heterogeneous wettability for unidirectionally self-pumping the sweat.) and flexible circuit board. Reproduced with permission [141]. Copyright 2024, Springer Nature

The human body maintains body temperature and water-salt balance through the secretion of sweat from the skin surface. However, prolonged wear of non-breathable electronic devices prevents the evaporation of water from the surface, which will lead to discomfort and even allergic reactions and inflammation. Furthermore, the secretion of sweat tends to disrupt the interaction between the electronics and the skin, which will affect its long-term stable operation. And non-breathable electronic devices resulting in the accumulation of sweat at the interface between the skin and devices will lead to disruption of the skin-device interaction and affect the long-term mechanoelectrical stability of the device’s operation. For achieving long-term health monitoring, it is necessary to improve the breathability of the on-skin electronics to promote the evaporation of water and sweat from the surface of human skin. It has been demonstrated that the desirable performance of a breathable electronic device is characterized by a water vapor transmission rate (WVTR) of higher than 20 and 1000 g m^−2^ h^−1^ at rest and during exercise, respectively [129]. One approach to enhance the breathability is constructing ultrathin, substrate-free electronic devices. For instance, Fang et al*.* constructed a free-standing, breathable submicron electrode (230 nm) with satisfactory gas permeability and ion permeability [130]. Another example is the preparation of a 150 nm thick stretchable electrode based on thermoplastic elastomer membrane by bubble blowing. The experimental results indicate that the electrode has excellent gas permeability (WVTR up to 580.18 g m^−2^ d^−1^), and it is observed that as the thickness of the TPE film increase, the gas permeability significantly decreases (Fig. 9a) [131].

As mentioned above, nanoscale ultrathin electronics have weak mechanical strength and are more difficult to fabricate. The utilization of porous materials in the fabrication of electronic devices represents another effective approach to achieving high breathability [132, 133]. As shown in Fig. 9b, Sun et al*.* transferred the patterned porous graphene sensing materials onto porous elastomeric sponge substrates to create multifunctional bioelectronic devices with notable breathability (with WVTR of 18 mg cm⁻^2^ h⁻^1^) [134]. Figure 9c presents a breathable electrode comprising porous substrates and conductive nanostructures. The porous structure is prepared using a straightforward respiratory mapping method and embedded with AgNWs as conductive materials, resulting in breathable (23 mg cm^−2^ h^−1^) and stretchable epidermal electrodes [135]. Owing to their tunable porosity, mechanical properties, and thickness, electrospun nanofibers have emerged as a prevalent material used in the fabrication of breathable epidermal electronics [136]. Figure 9d presents a local filtration approach to fabricate patterned metal nanowires (NWs) on electrospun thermoplastic polyurethane membranes (ENM), using porous carbon paper as the supporting substrates. Laser processing is utilized to enhance the bonding strength of NW-TPU fibrous membranes [137]. The process allows for the in situ preparation of nanofiber-based breathable electronics (432 g m^−2^ d^−1^), thereby contributing to the realization of integrated and personalized epidermal bioelectronics.

Despite excellent breathability, the fabrication of high-resolution patterned circuits on breathable nanofiber substrates is a significant challenge in the field of integrated bioelectronics. Zhuang et al*.* reported the development of ultrahigh-resolution nanofiber mat-based liquid metal patterning based on photolithography and water-transfer techniques, with feature sizes of up to 2 μm and densities of more than 75,000 electrodes cm^−2^ (Fig. 9e) [138]. The fabricated bioelectronics exhibit a high level of mechanical flexibility (up to 1000% strain), electrical conductivity (1.3 to 3.9 × 10^5^ S cm^−1^), and breathability (the air permeability and the moisture permeability reached 235 mm s^−1^ and 990 g m^−2^ d^−1^). Moreover, by integrating a breathable SBS fiber mat, liquid metal, and a bioadhesive hydrogel, Yao et al*.* developed a fully integrated breathable haptic textile system comprising a large-area and high-resolution haptic electrode array (~ 1 pixel cm^−2^ (overall) and 2.26 pixel cm^−2^ in the fingertips region) (Fig. 9f). This system demonstrates excellent breathability (the air permeability and the moisture permeability reached 40 mm s^−1^ and 657 g m^−2^ d^−1^), stretchability, and adhesion, and enables stable and excellent dynamic haptic feedback under both dry and wet conditions [139]. Another strategy for achieving breathability in epidermal electronics is microstructure design. Lan et al*.* developed a dry electrode with excellent breathability by combining a treefrog web-like structure with asymmetric conical holes (Fig. 9g). The asymmetric tapered pore structure is designed to achieve unidirectional transport of human sweat to avoid sweat accumulation, thus improving the permeability (40 ~ 82 mg cm^−2^ h^−1^) of the device [140]. Zhang et al*.* reported a three-dimensional liquid diode (3D LD) with spatially heterogeneous wettability, which was capable of spontaneously and rapidly transporting sweat unidirectionally from the skin-device interface to the outlet, demonstrating excellent air/sweat permeability (~ 70 g m^−2^ h^−1^). Moreover, the 3D LD can serve as a substrate for integration with wearable devices, thus enabling skin-integrated electronic systems with high breathability and providing reliable and comfortable biosignal monitoring (Fig. 9h) [141]. A corresponding quantitative comparison about adhesion strength, breathability metrics are shown in Table 2.

**Table 2:** Summary of adhesion strength and breathability performance of various materials and structural design strategies

Materials	Adhesion/Breathability Strategy	Adhesion Strength	Breathability property	Application	References
Au/Parylene	Ultrathin nanofilm (300 nm) by van der Waals	135.09 mN cm^−1^	N/A	EGG, EMG(10 h)	[115]
Au/PU-PDMS	Ultrathin nanofilm (165 nm)	159 μJ cm^−2^	N/A	1 week ECG	[116]
PAAm–alginate	Sub-10 µm thick hydrogel film	0.5 J m^−2^	1890.0 ± 134.4 g m^−2^ d^−1^	Coupling organic device with skin	[104]
Alginate-PAAm	Electrostatic interactions and hydrogen bonding	90 N m^−1^	N/A	EMG	[121]
PVA/PAA	Physical and covalent cross-linking	400 J m^−2^	N/A	Bioadhesive on wet tissue	[122]
rGO/PDMS	Hexagonal micro-patterned hierarchical architecture	26.8 J m^−2^ (dry), 23.9 J m^−2^ (moist)	N/A	ECG	[126]
Au/Si/conductive adhesive	Microneedle adhesive	Insertion into the skin (200 μm)	8.6 g m^−2^ h^−1^	EMG	[128]
AgNWs/SEBS	Ultrathin free-standing film (150 nm)	N/A	580 g m^−2^ d^−1^	EMG, vibration sensor	[131]
Go/silicone elastomer sponge	Porous structure	N/A	18 mg cm^−2^ h^−1^	EMG, ECG EEG, hydration sensor	[134]
AgNWs/TPU	Porous structure	N/A	23 mg cm^−2^ h^−1^	EMG, ECG, touch sensor	[135]
Ag@Au NWs/TPU	Electrospun nanofiber	N/A	432 g m^−2^ d^−1^	Epicardial signal recording, nonenzymatic biosensor	[137]
LM/hydrogel fiber mat/SBS	Electrospun nanofiber	25–80 kpa (wet)	~ 700 g m^−2^ d^−1^ (moisture) ~ 100 mm s^−1^ (air)	ICU-grade postoperative cardiac care	[95]
LM/PDMS	Web-like structure with asymmetric conical holes	10 kPa(dry), 5 kPa(wet)	40 ~ 82 mg cm^−2^ h^−1^	EMG, ECG, EEG	[140]
Au/Polyester fabrics/PDMS	Three-dimensional liquid diode	N/A	~ 70 g m^−2^ h^−1^	ECG, integrated weather station	[141]

### Mechanoelectrical Stability

The mechanoelectrical stability of on-skin electronics is a crucial guarantee for achieving continuous health monitoring. Mechanoelectrical stability encompasses a range of characteristics, including mechanical durability, electrical stability, skin-environmental resistance, and self-healing. For instance, the aging of functional materials (e.g., dopant leaching and oxidative degradation of conductive polymer, solvent evaporation and filler leakage of composite materials) and the influence of the skin environment (sweat, oils) may result in the gradual deterioration of device functionality, potentially leading to its ultimate failure. Furthermore, repeated strain from body motion may lead to microcracking, delamination within multilayer structures, or fatigue-induced failure of conductive paths. This is particularly relevant for systems that must endure thousands of cycles over days or weeks. Consequently, it is necessary to develop new materials, structural design strategies, or manufacturing methods to achieve mechanoelectrical stability for on-skin epidermal electronics.

**Fig. 10:**
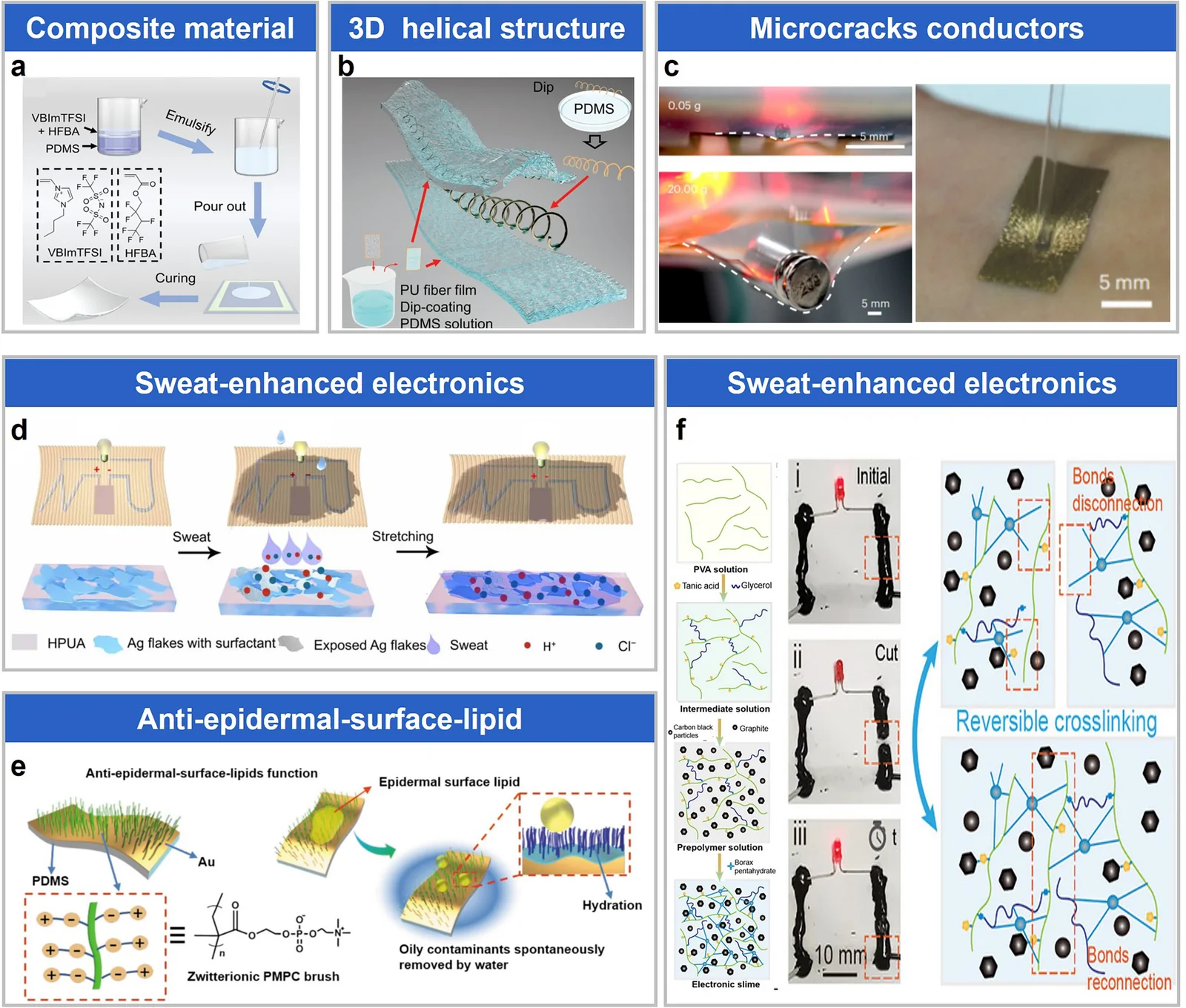
Strategies for realizing mechanoelectrical stability of on-skin electronics. **a** Long-term stable elastomer composites material for robust electronics. Reproduced with permission [142]. Copyright 2024, Wiley–VCH. **b** Lantern-inspired helical interconnects structure for on-skin epidermal electronics. Reproduced with permission [143]. Copyright 2023, Wiley–VCH. **c** Thermal expansion-induced microcracks conductors with excellent electromechanical performance for long-term stable and robust on-skin electrode. Reproduced with permission [41]. Copyright 2022, Springer Nature. **d** Schematic of the enhanced conductivity from human sweat and the preparation of the Ag-HPUA electrode. Reproduced with permission [144]. Copyright 2021, American Association for the Advancement of Science. **e** Configuration of the anti-lipid electrode and the water-enabled oil-cleaning effect. Reproduced with permission [145]. Copyright 2020, Wiley–VCH. **f** Self-healing electronic slime for durable epidermal wearable electronics. Reproduced with permission [147]. Copyright 2024, Wiley–VCH

For instance, Zhang et al*.* presented an elastomeric composite material with excellent stability through the copolymerization of ionic liquid monomer with fluorinated acylate (Fig. 10a). This composite material can maintain excellent electromechanical performance at high temperatures (250 °C) and after long-term (6 months) exposure at 80 °C [142]. Another strategy for achieving mechanoelectrical stability is to construct conductive network with large stretchability and high conductive stability, while maintaining their stable performance through rational encapsulation. Inspired by the accordion lantern with a hollow structure, Li et al*.* proposed a self-adhesive, tough epidermal electronics consisting of a metal fiber and two electrospun polymer fiber films, where the three-dimensional helical metal fiber acted as a highly stable conductor and the polyurethane (PU) fiber film served as a self-adhesive substrate and encapsulation. Similar to the robust bonding structure between the inner skeleton and the covering thin layer of lantern, the on-skin electronics possess excellent electrical stability (less than 0.5% electrical resistance change upon 100% elongation) and immunity to motion interference (Fig. 10b) [143]. In addition, through thermal expansion-induced microcracks technology, Jiang et al*.* reported a 1.3 µm thick stretchable PDMS-Au conductor with excellent mechanical durability and electrical property consistency, which exhibited 1.7% resistance increase at 0% strain after 5000 cycles at 100% strain. The excellent mechanoelectrical stability can attribute to the preformed microcracked surface, which prevents further damage during the stretchable process. Moreover, the on-skin electrodes constructed from this conductor possessed excellent self-adhesive, breathable, and waterproof properties (Fig. 10c) [41].

As previously stated, perspiration has an impact on the electrical stability and interfacial bonding strength of electronic devices. In order to circumvent the effects of perspiration on epidermal electronics, researchers have primarily attempted to either design highly permeable structures or utilize surface encapsulation. It is noteworthy that Lv et al*.* propose an alternative approach, whereby flexible electronics are not only unaffected by sweat, but rather exhibit enhanced performance due to the presence of sweat. They developed a stretchable silver electrode comprising a conductive silver flake and an elastic binder (the thermoplastic and hydrophilic poly(urethane–acrylate) (HPUA)), which had been demonstrated that the conductivity was not only unimpaired by sweat but could in fact be enhanced through the synergistic sintering effect of lactic acid and Cl^−^ present in sweat (Fig. 10d) [144]. This method offers a novel approach to the preparation of long-term stable and stretchable bioelectronics. Apart from sweat, skin oil and grease can also affect the sensing performance of epidermal electronics. However, there is a relative lack of research on the anti-oil function. By grafting zwitterionic polymer brushes onto the stretchable gold-coated poly(dimethylsiloxane) (Au/PDMS) surface, He et al*.* developed an electrode with self-cleaning properties for removing grease in an aqueous environment. The grafted superhydrophilic zwitterionic brushes exhibit oil-repellent properties in aqueous environments, enabling the removal of oil and grease from the electrode surface through water rinsing without compromising the electrode’s performance (Fig. 10e) [145]. Mechanoelectrical stability of epidermal electronics can also be achieved by constructing flexible devices with self-healing capabilities [146]. Figure 10f illustrates a super-deformable (2600% strain), electromechanically durable, and strongly self-healing (fastest recovery time ≈ 1 s, maximum wound distance ≈ 5 mm) wearable E-slime. The self-healing capacity of the material is primarily attributable to the formation of dynamic catechol-borate bonds between tannic acid and borax, as well as supramolecular interactions between tannic acid, PVA, and glycerol [147].

## Applications of On-Skin Electronics for Health Monitoring

The human body continuously generates numerous physical and electrical physiological signals that are closely associated with health issues. These signals can be continuously monitored by means of on-skin electronics attached to the skin, which enables the assessment of an individual’s health status, the early diagnosis of diseases and the monitoring of medical rehabilitation. This section will discuss the application of epidermal electronics to the long-term monitoring of electrophysiological signals and physio-physiological signals.

### Biophysical Signals

Biophysical signals including mechanical physiological signals and temperature signals. The monitoring of biophysical signals of the human body can be employed in the fields of disease prevention and diagnosis, patient rehabilitation, and elderly care. Mechanical physiological signals can be broadly classified into two categories: large-scale movements and subtle movements. Large-scale movements such as walking and exercising can provide immediate insight into the body’s movement status, offering valuable information about human health. For instance, regular analysis of body movements can reveal abnormal gait patterns and sudden hand tremors, which indicate underlying pathology such as Parkinson’s disease and Alzheimer’s disease [148]. Subtle movements such as respiration, heart rate, blood pressure, and vocalizations can also be a viable source of data for health assessment and contribute to the prevention, diagnosis, and treatment of diseases, particularly cardiovascular disease [32, 149]. This section outlines the application of epidermal electronics to different parts of the human body, with a view to achieving continuous and accurate monitoring of human biophysical signals. Moreover, body temperature is also a significant indicator of an individual’s health status. Installing temperature sensors on human skin enables continuous monitoring of body temperature fluctuations, which can assist in the prevention and diagnosis of diseases.

**Fig. 11:**
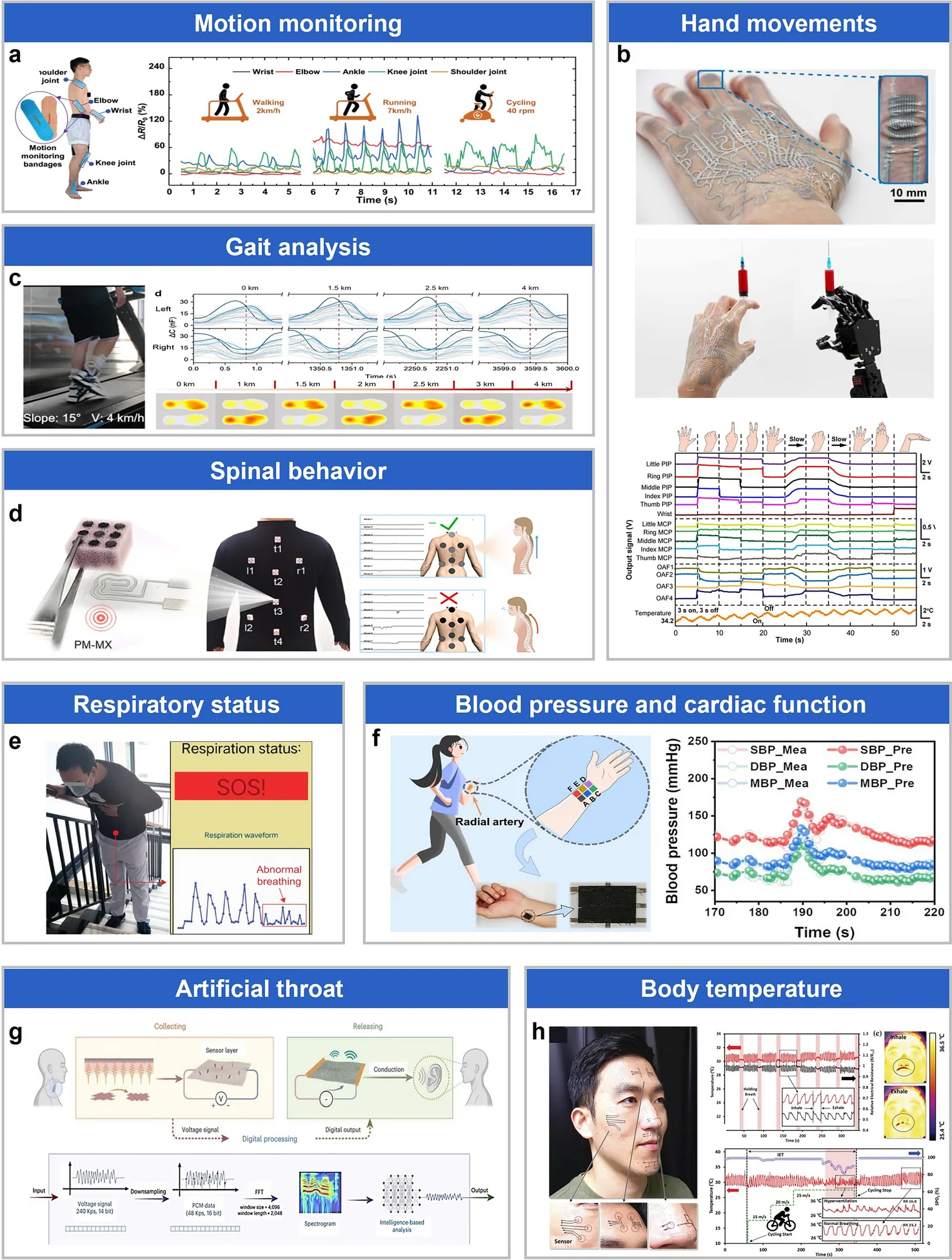
On-skin epidermal electronics for physio-physiological recording. **a** Epidermal Fabric Strain Sensors for motion monitoring for sports. Reproduced with permission [150]. Copyright 2023, Wiley–VCH. **b** On-skin METT for real-time recoding of different hand movements. Reproduced with permission [152]. Copyright 2021, American Association for the Advancement of Science. **c** Integrated iontronic flexible pressure sensor for human plantar pressure and gait analysis. Reproduced with permission [153]. Copyright 2024, American Chemical Society. **d** Dual network piezoresistive sensor for spine behavior monitoring. Reproduced with permission [156]. Copyright 2024, Elsevier. **e** Wearable healthcare system based on fiber strain sensors for monitoring respiratory status. Reproduced under terms of the CC-BY license [158]. Copyright 2024, National Science Review. **f** Wearable strain sensor array for real-time blood pressure and cardiac function monitoring. Reproduced with permission [159]. Copyright 2023, American Association for the Advancement of Science. **g** Speech interaction paradigm based on the wearable artificial throat. Reproduced with permission [160]. Copyright 2023, Springer Nature. **h** Conformal epidermal temperature sensor for respiration sensing. Reproduced under terms of the CC-BY license [162]. Copyright 2019, Wiley–VCH

Bai et al*.* proposed a strain fabric sensor with adjustable sensitivity, which was developed by electroplating a thin layer of gold with a defined fiber arrangement on a nanofiber mat in a pre-stretched state. (Fig. 11a) [150]. Researchers have found a negative correlation between grip strength and walking pace with the risk of developing Parkinson’s disease [151]. The strain sensor demonstrates excellent mechanical flexibility, enabling the monitoring of walking speed. When utilized in conjunction with epidermal electrodes that can assess grip strength in the human hand [77], it has the potential to serve as a screening tool for individuals at risk for Parkinson’s disease. Tang et al*.* created a multilayer integrated LM electronic tattoo with crease amplification that could conformally attach to human skin. By employing a layer-by-layer fabrication strategy, the researchers integrate 15 strain sensors into METT, which is capable of measuring 15 degrees of freedom of the hand (Fig. 11b) [152]. This capability provides invaluable feedback during rehabilitation exercises for Parkinson’s disease patients, allowing therapists and patients to objectively track subtle improvements in movement amplitude, speed, and coordination that might be imperceptible to the naked eye, thereby facilitating personalized training adjustments and motivation. Li et al*.* developed an integrated pressure sensor system consisting of polyimide and ionic fibers, which could achieve a self-adhesive interface between the ionic fibers and the functional layer by controlling the aggregation structure of the PI (Fig. 11c) [153]. Furthermore, by integrating the sensor output with a 1D convolutional neural network (1D-CNN), various plantar pressure distribution maps can be classified, achieving a recognition accuracy of 99.8%. The system exhibits high sensitivity and reliability, enabling precise detection of plantar pressure distribution and gait analysis, which is a valuable diagnostic tool that can be utilized to understand the relationship between motor and cognitive functions in older adults. This analysis has the potential to predict the progression of motor and cognitive disorders and offers a significant opportunity for the early identification and assisted diagnosis of Parkinson’s and Alzheimer’s disease [154, 155]. Inspired by the structure of human column epithelial cells, Ren et al*.* proposed an efficient heterogeneous assembly strategy for the development of a dual-network piezoresistive sensor (PM-MX) based on polyurethane (PU), melamine (MA) sponge and MXene nanosheets with ultrahigh sensitivity. (Fig. 11d) [156]. Based on the PM-MX sensor and deep learning algorithms, a dorsal domain sensor network is constructed for monitoring human spinal behavior, which can provide real-time warning of poor sitting posture and prevent spinal overload and potential injury or disorders caused by incorrect posture [157].

YU et al*.* described a high-sensitivity fiber strain sensor that adhered functional materials in situ to the fiber surface through a hydrolytic condensation process, forming a sensing layer with strong interfacial adhesion. This method enables the sensor to be more susceptible to cracking and suitable for integration into textiles to develop wearable healthcare systems. The system is capable of detecting and precisely recording a wide range of respiratory conditions, offering significant potential for asthma monitoring and real-time alerting (Fig. 11e) [158]. In order to achieve real-time and long-term monitoring of blood pressure and cardiac function, Li et al*.* developed a smart blood pressure and cardiac function monitoring system based on a conformal strain sensor array. The integrated wearable system, which combined pulse data collected by six high-performance strain sensors with a deep learning neural network, is capable of monitoring blood pressure and heart status in real time (Fig. 11f) [159]. Speech represents a crucial mode of human communication. However, the speaker’s state of health (e.g., voice impairment resulting from stroke, cerebral palsy, trauma, etc.) and the surrounding environment (noise interference) frequently impact the transmission and recognition of sound. In light of these considerations, Yang et al*.* developed a wearable artificial throat (AT) that was capable of speech perception and vocalization (Fig. 11g) [160]. The AT forms conformal contact with the skin and detects a multimodal signal composed of low-frequency muscular movements and surface-transmitted acoustic vibrations. To accurately recognize these complex speech-related signals, the authors employed an improved hybrid deep learning model that integrates AlexNet, ReliefF, and SVM, achieving a speech recognition accuracy of 91%. Based on the recognized text, a text-to-speech (TTS) synthesis module was used to generate artificial vocal signals, which were then fed back to actuate the AT. This bidirectional system enables functional voice restoration for individuals with speech impairments. Body temperature is another typical vital sign indicative of an individual’s health status, and precise measurement can yield critical insights for health monitoring [161]. Shin et al. proposed a one-step monolithic laser reduction sintering process for fabricating high-performance temperature sensors suitable for human skin for long-term physiological temperature sensing (Fig. 11h). The patterning of Ni-NiO–Ni heterostructures is achieved by a laser direct writing process, and the temperature sensors prepared from them had low thickness and low stiffness. This design enables seamless integration with human skin, facilitating real-time capture of respiratory signal variations. Alterations in respiratory characteristics often accompany various life-threatening metabolic or pathological imbalances, such as acidosis, alkalosis, and sepsis. Consequently, the epidermal temperature sensor exhibits significant potential for application in the non-invasive monitoring of critically ill patients [162]. Furthermore, the ambient-compatible, high-speed, and energy-efficient nature of laser sintering renders it particularly well-suited for scalable and cost-effective manufacturing of large-area flexible electronics.

### Electrophysiological Signals

As one of the most crucial vital signs, the long-term recording of human biopotentials permits the early detection, diagnosis, and recovery from various diseases related to brain, heart, and muscle, hence offering significant practical value in health monitoring. This section discusses the typical electrophysiological signals monitored using different on-skin epidermal electronics, including ECG, EMG, and EEG.

#### ECG

**Fig. 12:**
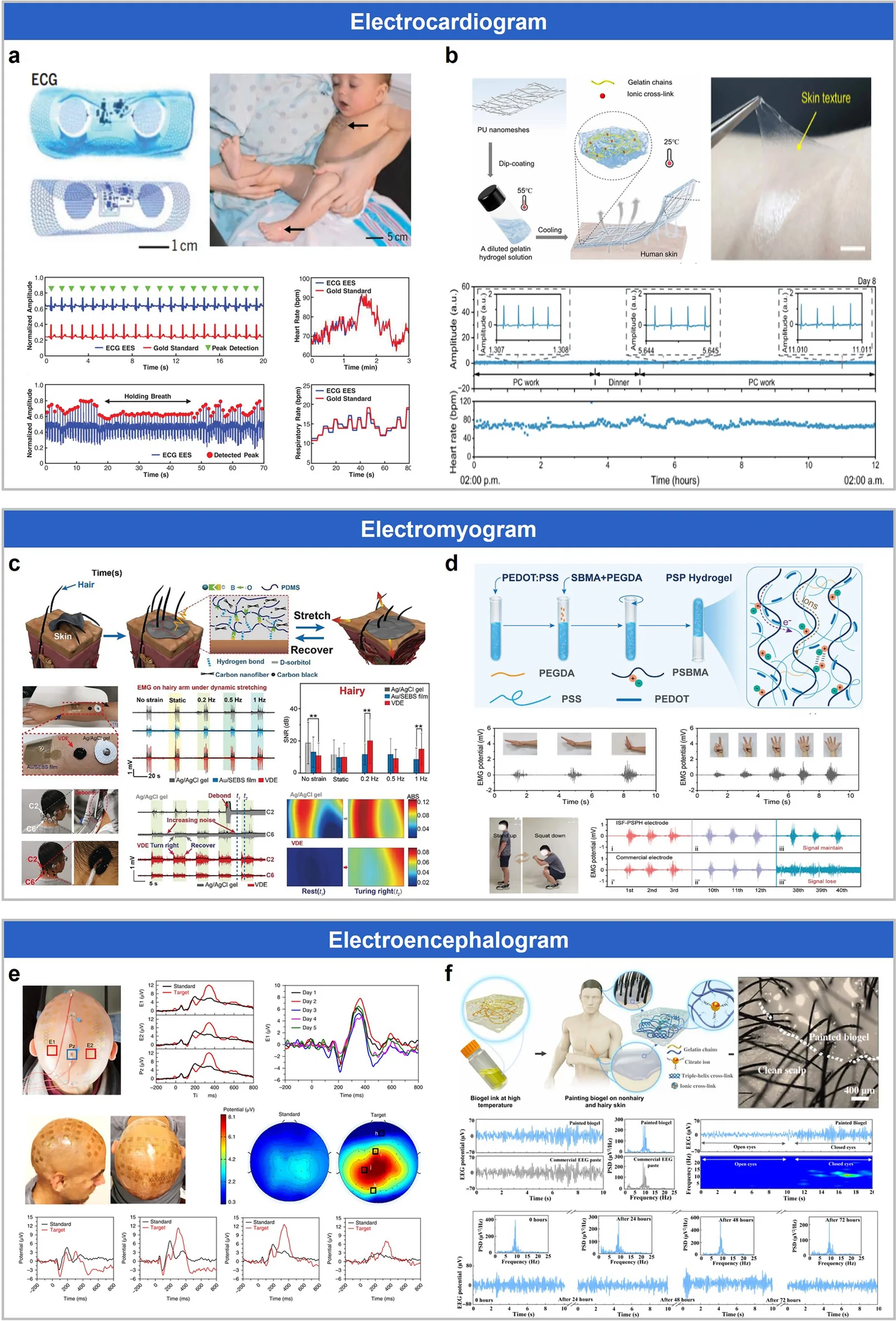
On-skin epidermal electronics for electrophysiological recording. **a** Wireless epidermal electronic system for neonatal intensive care. Reproduced under terms of the CC-BY license [164]. Copyright 2019, American Association for the Advancement of Science. **b** Conformal wet hydrogel electrode for long-term ECG monitoring. Reproduced with permission [165]. Copyright 2024, American Association for the Advancement of Science. **c** Hairy-skin-adaptive viscoelastic dry for stable dynamic EMG recoding. Reproduced with permission [170]. Copyright 2023, Wiley–VCH. **d** In situ forming hydrogels electrophysiological electrodes to detect sEMG signals. Reproduced with permission [171]. Copyright 2023, Wiley–VCH. **e** Large-area epidermal electrodes for multichannel EEG. Reproduced with permission [177]. Copyright 2019, Springer Nature. **f** On-skin paintable biogel interface for high-fidelity and long-term EEG recording. Reproduced with permission [20]. Copyright 2022, American Association for the Advancement of Science

As one of the most prevalent forms of biopotential analysis, ECG reflects the electrical impulses generated by the heartbeat, offering a comprehensive overview of cardiac health and functioning. Through the interpretation of electrocardiographic tracings, medical professionals are able to screen for a wide range of cardiac conditions, including arrhythmias, myocardial infarction, structural abnormalities, and various cardiovascular diseases [163]. Epidermal electronics utilized for the monitoring of electrophysiological signals, including ECG, EMG, and EEG, are generally categorized into two types: flexible dry electrodes and wet electrodes. Dry electrodes are considered to be a wearable electrode suitable for prolonged detection of bioelectrical signals due to their durability, portability, and biocompatibility. However, the poor skin-electronics interface between dry electrodes and the skin, particularly during body movement and skin sweating, results in significant signal noise and motion artifact, which limits the practical application of dry electrodes. The preparation of dry electrodes with reliable adhesion can be achieved through the selection of appropriate materials and the implementation of a reasonable structural design, as detailed in “3.1. Adhesiveness.” The present subsection is dedicated to the discussion of the application of on-skin epidermal electronics for the acquisition of ECG signals. For instance, Chuang et al*.* reported an ultrathin, ultrasoft wireless epidermal electronic system (EES) for neonatal intensive care (Fig. 12a). The EES was composed of filamentary metal mesh microstructures exhibiting fractal geometry, which could be directly adhered to the chest and feet of newborns, without multiple wires connected to rigid sensors. The integrated and wireless EES allow for the gentle, continuous, and noninvasive monitoring of ECG signals for highly vulnerable babies in intensive care units, exhibiting excellent performance on par with the most sophisticated clinical-standard monitoring devices [164].

Flexible wet electrodes are typically composed of flexible, biocompatible hydrogels that are capable of achieving conformal contact with the skin. The modulus of the hydrogel wet electrode is similar to that of the skin, which effectively minimizes the mechanical mismatch and enable long-term stable physiological signal monitoring. A discussion of hydrogel epidermal electronics can be found in subsection “2.5 Hydrogel,” where the potential for improving the mechanoelectrical stability of the gel is explored. To further improve the performance of the wet electrodes, various conductive materials, including PEDOT:PSS, graphene, MXene, salt ions, and ionic liquids are incorporated into the hydrogel to enable the preparation of hydrogel bioelectrodes with excellent electrical properties for long-term ECG signal acquisition. Wang et al*.* proposed an ultrathin, breathable, and self-adhesive nanofiber-reinforced hydrogel electrode by immersing polyurethane nanofibers in a conductive gelatin solution (Fig. 12b). Attribute to the sodium sulfate additive, the nanofibers reinforcement strategy, and the reversible thermal-dependent phase transition of gelatin, the bioelectrode exhibits excellent electrical properties, mechanical robustness, adhesion, breathability, and is capable of prolonged (> 1 week) ECG signal monitoring, demonstrating great potentiality for early diagnosis of cardiac disorders [165]. Recently, artificial intelligence (AI), including machine learning and deep learning, has been used in healthcare and medicine to achieve more robust and automated signal interpretation. With the help quick and accurate interpretation of images, AI is expected to enhance diagnostic accuracy, making healthcare more efficient and cut costs. For instance, by enabling continuous, real-time signal processing, AI facilitates early detection of cardiovascular abnormalities such as arrhythmias and heart failure, allowing for timely medical intervention [166].

#### EMG

Electromyography can be used to discern and analyze the biopotentials of human muscles, particularly suitable for diagnosis and recovery of patients suffering from stroke, myasthenic syndrome, Parkinson’s disease, etc. [167–169]. Epidermal electrodes are typically positioned on human facial, arm, or leg muscles for non-invasive monitoring, which means that a stable, adhesive bioelectronic interface is essential to avoid motion artifacts. Tian et al*.* presented a hairy-skin-adaptive viscoelastic dry electrode comprising a conductive carbon material and sorbitol-modified polyborosiloxane. The dry bioelectrode displays liquid-like behavior at low shear rates and is capable of penetrating hairs and filling in wrinkles in the skin, thereby achieving seamless contact and minimizing motion artifacts (Fig. 12c). In comparison to commercial gel electrodes, the dry electrode demonstrated superior signal-to-noise ratios at varying dynamic deformation frequencies, indicating its exceptional resilience to dynamic deformation and capacity to record EMG signals with precision and stability [170]. Figure 12d displays the fabrication of a self-adhesive conductive gel based on PEDOT:PSS-promoted spontaneous polymerization of amphiphilic ionic polymers, which exhibits notable elasticity, conductivity, and biocompatibility. The hydrogel electrode can be fabricated in situ on human skin, which enables it to maintain close contact with the hairy dermis. It is observed that the electrode is able to accurately discriminate the EMG signals generated by different wrist bending angles and different hand gestures [171]. Beyond signal acquisition, EMG data processed using deep learning have demonstrated strong potential for neurological rehabilitation and human–machine interaction. For example, domain-adaptive learning frameworks such as EMGSense achieved 91.9% cross-subject gesture recognition accuracy, significantly improving the scalability of wearable systems in gesture control and rehabilitation engineering [172].

#### EEG

Electroencephalography is a recording of electrical potentials associated with brain activity. Long-term EEG monitoring provides valuable information for the diagnosis and treatment of neurological disorders, like status epilepticus diagnosis [173], sleep patterns monitoring [174], vigilance and cognitive states estimating [175]. Due to its high temporal resolution, mobility, and relatively low cost, EEG non-invasive monitoring of brain electrical activity plays an important role in clinical settings and brain-computer interface research [176]. In comparison to other bioelectrical signals, such as ECG and EMG, EEG signals are relatively weak (microvolt amplitude), and the main frequency range is above 0.3–30 Hz, which makes it more challenging to achieve efficient EEG recording. Conformal contact between electrodes and the scalp surface is essential for continuous and stable monitoring of electrophysiological signals with high signal-to-noise ratios. However, thick hair can interfere with the stability of the electrode-scalp interface. Figure 12e presents electronic tattoos for large-area whole-head EEG recording. A distinctive open mesh structure with elastomeric bilayer designs and removable polymer supports is devised with the objective of reducing the risk of heating and electromagnetic interference. The electronic tattoo can be used to chronic recordings (five days to two weeks) without interfering with normal daily activities [177]. In order to achieve reliable contact, Wang et al*.* developed a biocompatible conductive biogel, which was directly coated on the skin surface by temperature-controlled phase change transformation to form a stable conformal contact between the electrode and the hair scalp (Fig. 12f). This enables high-fidelity recording of EEG signals for three consecutive days, which has the potential to be applied in the field of long-term monitoring [20]. EEG signals have shown significant value in the objective assessment of mental health conditions, particularly depression—a major global public health concern. Early detection and intervention are critical for preventing symptom escalation, yet existing diagnostic methods often lack accessibility and objectivity. With the development of AI algorithms, EEG-based depression screening is becoming increasingly feasible. For instance, EEG signals collected from frontal regions (Fp1, Fpz, Fp2) have been processed using various machine learning classifiers, including SVM, k-NN, and Random Forest, achieving reliable differentiation between depressed and non-depressed individuals [178]. Furthermore, deep learning models such as multilayer perceptron (MLPs), deep belief network (DBNs), and long-short term memory (LSTM) have also been applied to EEG data from frontal and occipital lobes, enabling automatic feature learning and robust classification performance. These approaches highlight the emerging potential of wearable EEG systems, combined with machine intelligence, for scalable and objective diagnosis of affective disorders such as depression [179].

## Conclusion and Outlook

**Fig. 13:**
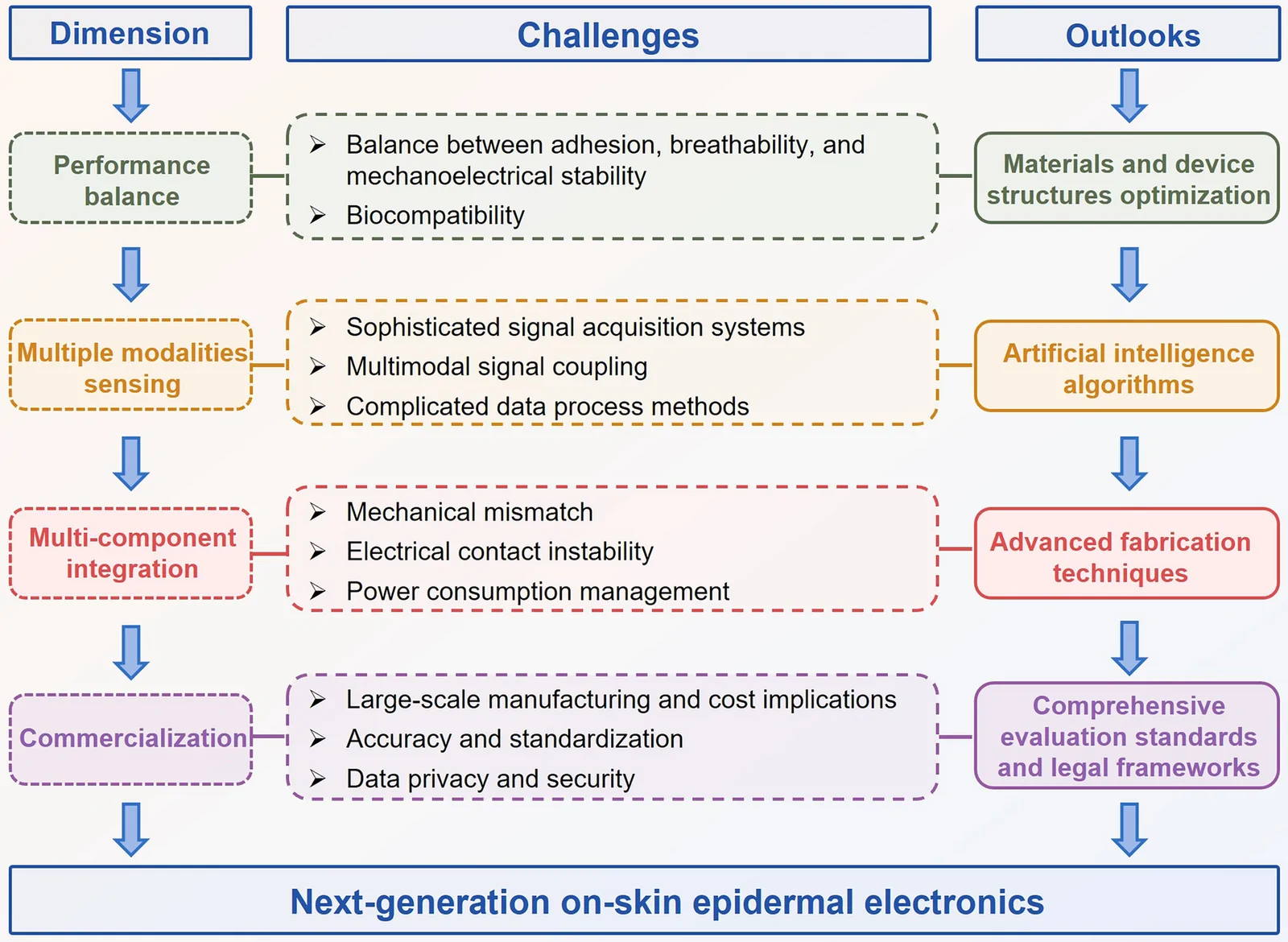
Challenges and outlooks of the next-generation on-skin epidermal electronics

The utilization of advanced wearable on-skin epidermal electronics has facilitated the continuous monitoring of a range of diseases and enabled the implementation of personalized health management, disease early prevention, as well as timely diagnosis and patient rehabilitation. In this review, we present a comprehensive overview of recent advancements in on-skin epidermal electronics. The material and structure design suitable for constructing high-performance on-skin epidermal electronics and the desired device performance required to achieve long-term and continuous health monitoring are discussed comprehensively. A systematic analysis and discussion of methods and strategies for realizing epidermal electronics with robust self-adhesion, excellent breathability, and prolonged electromechanical stability that can be tightly adhered to human skin for long-term and continuous health monitoring are presented. The healthcare applications of advanced on-skin epidermal electronics in biophysical and electrophysiological signal monitoring, like spinal disorders prevention, blood pressure monitoring, and neonatal intensive care, are also reviewed. Notwithstanding the considerable advancements that have been made in this field, several challenges remain that warrant further development and widespread adoption of on-skin epidermal electronics (Fig. 13).

One of the primary challenges in the development of epidermal electronics lies in the synchronous realization of several key properties: conformability, self-adhesiveness for seamless and robust skin-device interface, breathability for comfortable wear, and stable mechanoelectrical performance for prolonged use. Continued efforts are required to optimize materials and device structures to achieve a balanced integration of adhesion, breathability, and electromechanical stability. To further accelerate and systematize this optimization process, AI, particularly machine learning (ML), is emerging as a powerful enabler. By extracting complex correlations from large datasets encompassing material composition, fabrication conditions, and device performance, ML algorithms can assist in predicting optimal material combinations, tuning processing parameters, and uncovering structure–property relationships that may otherwise be overlooked. In addition, AI-guided design frameworks may facilitate the development of personalized, adaptive epidermal systems tailored to specific physiological signals or user conditions, thereby bridging the gap between laboratory innovation and clinical translation.

For skin-interfaced devices aimed at high-precision physiological signal acquisition, as biological signals are inherently ionic, future material designs must move beyond traditional purely electronic conductors toward ion–electron mixed conduction systems. Organic conductive polymers such as PEDOT:PSS, which intrinsically facilitate both ionic and electronic transport, represent a highly promising platform. Enhancing these materials through ionic doping (e.g., functionalized ionic liquids responsive to muscle-specific metabolites like lactate and creatine kinase for selective enhancement of muscle activity–related signals), blending with ion gels, biocompatible polymers (e.g., peptide-based polymers), or nanocomposite engineering (e.g., synergistic integration of nanoscale liquid metal microemulsions to establish multiscale mixed conduction networks) can substantially reduce the electrode–skin interface impedance and improve signal fidelity and stability. Moreover, the advancement of epidermal electronics may benefit from bioinspired structural designs. In particular, fractal architectures, with their hierarchical and self-similar properties, provide improved strain distribution, fault tolerance, and enhanced stretchability compared to traditional serpentine or mesh patterns. These structures hold significant potential for conformal integration in highly dynamic body regions.

The biocompatibility of epidermal electronics is also a pivotal factor in determining its suitability for long-term monitoring applications. The potential toxicity of certain conductive functional materials may result in skin irritation and inflammation, particularly under prolonged contact. To address this, non-toxic and biocompatible conductive networks can be developed by employing biocompatible materials (e.g., PEDOT:PSS and gold), or through material optimization strategies, such as molecular engineering or blending multiple materials. However, there is still room for improvement with regard to the mechanical properties of current biocompatible materials. A promising approach involves combining them with synthetic polymers to enhance both biocompatibility and durability.

The integration of multiple sensing modalities has demonstrated considerable potential for enhancing decision-making in health monitoring and medical applications. In scenarios necessitating multimodal data acquisition from a single body region, such as the measurement of skin hardness through the integration of strain and pressure sensors, the prevailing multimodal sensors are predominantly fabricated by stacking or arranging diverse functional materials in parallel to collect a variety of biological signals. This approach necessitates the implementation of sophisticated signal acquisition systems. An effective approach involves the utilization of diverse sensing mechanisms of a single sensor to acquire multiple signals. For scenarios where signals need to be captured from multiple body regions (e.g., ECG, EMG, and EEG signals from the chest, target muscle region, and head, respectively), a multiregional data acquisition network can be engineered by distributing epidermal electronic devices that possess distinct signal-acquisition functionalities in the corresponding body parts. However, it should be noted that both multimodal data from a single region and multiple signals from multiple regions will inevitably lead to signal coupling problems. Machine learning-driven data analysis is poised to emerge as a promising solution to address these challenges. The employment of artificial intelligence algorithms facilitates the effective separation of multimodal data acquired by flexible sensors, enabling the fusion and analysis of multiple physiological signals, ultimately resulting in a comprehensive health status assessment and accurate diagnostic decisions.

Multi-component integration, including front-end epidermal electronics, wireless communication components, data processing units, storage modules, and power supply modules, represents another significant challenge in the development of advanced and practically applicable wearable electronics. Currently, a plethora of commercial chips and power supply systems are available to meet the integration requirements of epidermal electronics. A potential solution for achieving integrated wearable systems lies in the combination of mature and efficient integrated circuit chips with flexible electronics. However, the integration of these components into a single chip patch remains a considerable challenge, primarily due to the incompatibility between flexible devices and rigid modules, which leads to mechanical mismatch and electrical contact instability. Consequently, there is an imperative for developing advanced fabrication techniques that facilitate the integration of rigid and flexible components without compromising their performances.

Furthermore, effective power consumption management is crucial for ensuring the long-term functionality of wearable health monitoring systems. In this regard, the exploration of energy harvesting technologies, such as triboelectric nanogenerators (TENGs) and biofuel cells, along with high-efficiency battery designs, such as supercapacitors, is essential for ensuring a sustainable power supply in these devices. Among these strategies, triboelectric nanogenerators have attracted significant attention due to their ability to convert low-frequency biomechanical motions into electrical energy. Their high voltage output, material versatility, and compatibility with flexible substrates make them particularly suitable for integration into epidermal systems. However, practical application of TENGs in on-skin electronics still faces challenges such as limited current output, load matching issues, and environmental sensitivity (e.g., humidity and sweat). Future TENG-based systems should focus on enhancing power density through micro/nanostructured interfaces, charge management layers, and hybridization with complementary harvesting mechanisms. It should be note that future on-skin electronic systems need to embrace a co-design paradigm to address the interface compatibility between energy harvesting modules and skin-interfacing sensors, ensuring stable power delivery without compromising sensing accuracy or comfort.

To speed up the commercialization of epidermal electronics, several issues need to be noticed. Primarily, the feasibility of large-scale manufacturing and the associated cost implications must be thoroughly assessed. Despite the substantial progress made in epidermal electronics research in the laboratory setting, their large-scale production remains constrained by technical and economic limitations. Furthermore, the inherent thin and soft characteristics of epidermal electronics pose challenges in terms of reuse. Therefore, the realization of a low-cost, scalable manufacturing model is imperative for promoting the widespread use of epidermal electronics. In addition, most existing epidermal electronics require a transfer process to be applied to human skin, which has the potential to result in performance degradation or structural damage. The development of highly conductive and biocompatible conductive inks is expected to enable the direct printing of epidermal electronics on human skin, thereby promoting the efficient and cost-effective application of these devices. Another critical challenge pertains to the accuracy and standardization of epidermal sensors. To ensure the efficacy of epidermal electronic systems in real-world medical health monitoring, it is crucial to establish comprehensive sensor evaluation standards that ensure the sensors meet requisite levels of accuracy, stability, and repeatability across various scenarios. Finally, as epidermal electronics continue to gain prominence in health monitoring and other sectors, concerns pertaining to data privacy and security assume particular significance. In addition to technical safeguards, legal and ethical frameworks must be established to ensure transparency in data processing and legitimate protection of user privacy.

Despite the unresolved issues, it is anticipated that as the aforementioned challenges are addressed, multi-functional and intelligent on-skin epidermal electronics will be developed and implemented in everyday life. This “second skin” characteristic not only significantly enhances wearable comfort and compliance, providing unprecedented convenience and data continuity for long-term health monitoring and chronic disease management (such as cardiovascular diseases, diabetes, and neurological disorders), but also enables the sensitive detection of subtle physiological changes. This capability provides crucial data support for early disease warning, precision diagnosis, and the formulation of personalized treatment strategies. Furthermore, flexible on-skin epidermal electronics demonstrate significant potential in areas such as transdermal drug delivery, closed-loop therapeutic feedback systems, and dynamic assessment of post-operative rehabilitation and functional recovery. Their development is expected to profoundly revolutionize clinical monitoring practices, driving medical practice toward greater precision, personalization, remoteness, and prevention. This will facilitate long-term, continuous, and comfortable monitoring of human health signals, personalized healthcare management, offering a precise and efficient approach for the prevention, early diagnosis, and treatment of major diseases.
